# G6PC Downregulation Promotes Renal Calcium Oxalate Stone Formation via Lactate‐Induced SNAIL1 K206 Lactylation and Epithelial‐Mesenchymal Transition

**DOI:** 10.1002/advs.75585

**Published:** 2026-05-10

**Authors:** Kai Liu, Boming Zhang, Ruixin Fan, Chen Duan, Yangjun Zhang, Huahui Wu, Xiangyang Yao, Xiongmin Mao, Bo Li, Youmiao Zeng, Zhenzhen Xu, Yang Wang, Mengcheng Luo, Xinyu Xu, Ninghan Feng, Heng Li, Hua Xu

**Affiliations:** ^1^ Department of Urology Zhongnan Hospital of Wuhan University Wuhan Hubei China; ^2^ Department of Urology The First Affiliated Hospital of Shandong First Medical University & Shandong Provincial Qianfoshan Hospital Jinan Shandong China; ^3^ Shandong Medicine and Health Key Laboratory of Organ Transplantation and Nephrosis Shandong Institute of Nephrology Jinan Shandong China; ^4^ Department of Urology Tongji Hospital of Tongji Medical College Huazhong University of Science and Technology Wuhan Hubei China; ^5^ Tumor Precision Diagnosis and Treatment Technology and Translational Medicine Hubei Engineering Research Center Zhongnan Hospital of Wuhan University Wuhan Hubei China; ^6^ Department of Radiation and Medical Oncology Zhongnan Hospital of Wuhan University Wuhan Hubei China; ^7^ Department of Urology the First Affiliated Hospital of USTC Division of Life Sciences and Medicine University of Science and Technology of China Hefei Anhui China; ^8^ Department of Urology The First Affiliated Hospital of Shenzhen University Shenzhen Second People's Hospital Shenzhen China; ^9^ Department of Urology Jiangnan University Medical Center Wuxi Jiangsu China; ^10^ School of Basic Medicine Science Wuhan University Wuhan Hubei China; ^11^ Taikang Center for Life and Medical Sciences Wuhan University Wuhan Hubei China

**Keywords:** epithelial‐mesenchymal transition, glucose metabolism, kidney stone, lactylation, SNAIL1

## Abstract

Nephrolithiasis is increasingly prevalent worldwide. Although metabolic dysregulation has emerged as an important feature of the disease, the specific metabolic programs within tubular epithelial cells that drive stone formation remain poorly understood. Here, we use single‐cell transcriptomics to profile mouse models of nephrolithiasis and identify a marked suppression of gluconeogenesis in renal proximal tubular epithelial cells, driven by downregulation of the rate‐limiting enzyme glucose‐6‐phosphatase (G6PC) and associated with lactate accumulation. We further show that loss of G6PC or lactate accumulation is sufficient to activate TGF‐β/SMAD3 signaling, driving epithelial–mesenchymal transition (EMT) and a profibrotic epithelial program associated with enhanced crystal deposition. Mechanistically, lactate induces lactylation of the transcription factor SNAIL1 at lysine 206, facilitating its nuclear localization and transcriptional activation of TGF‐β. Together, these findings establish a G6PC‐lactate‐SNAIL1 axis linking metabolic dysregulation to epithelial remodeling during nephrolithiasis.

## Graphical abstract

In renal calcium oxalate stone formation, G6PC downregulation leads to lactate accumulation. This lactate mediates CBP/p300‐dependent lactylation of SNAIL1 at K206, promoting its nuclear translocation. Nuclear SNAIL1 activates the TGF‐β/SMAD3 pathway, driving epithelial‐mesenchymal transition and fibrosis, which ultimately facilitates crystal deposition and disease progression.

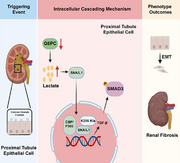

## 1 Introduction

The global incidence and recurrence of urinary stones disease continue to rise, presenting a major clinical challenge. Persistent nephrolithiasis can lead to urinary tract obstruction and chronic tubular injury, which may progress to interstitial fibrosis and glomerulosclerosis [1, 2]. In addition to these clinical complications, kidney stone disease substantially reduces quality of life and creates a considerable socioeconomic burden. Although current interventions, including extracorporeal shock wave lithotripsy, ureteroscopic lithotripsy, and percutaneous nephrolithotomy, achieve high short‐term stone clearance rates, they fail to address the root causes of stone formation. Consequently, postoperative recurrence remains common, with 5‐year recurrence rates approaching 55% [1]. These limitations highlight the need for preventive strategies that target the mechanisms of stone formation.

Kidney stone formation is a complex process that involves physicochemical, cellular, and systemic factors. Calcium oxalate (CaOx) stones account for approximately 75% of all renal calculi, with calcium oxalate monohydrate (COM) being the most common crystalline form [3]. Accumulating evidence suggests that nephrolithiasis represents a systemic metabolic disorder associated with metabolic dysregulation and sedentary behavior, rather than a purely localized renal condition [4]. However, the molecular mechanisms by which altered systemic metabolism promotes CaOx stone formation are not fully understood.

The kidney occupies a unique role in systemic glucose metabolism, with pronounced cell‐type specificity. Proximal tubular epithelial cells (PTECs) serve as the primary sites of both glycolysis and gluconeogenesis, whereas the thick ascending limb of the loop of Henle relies predominantly on glycolysis for energy. PTECs are enriched in mitochondria and peroxisomes, reflecting their high energy demand and central role in renal metabolic regulation [5]. Under pathological conditions, renal metabolic reprogramming is a hallmark of chronic kidney disease (CKD). Suppression of renal gluconeogenesis, accompanied by reduced glucose production and increased lactate accumulation, correlates with adverse renal outcomes [6]. Previous studies have shown that high glucose conditions upregulate osteopontin (OPN) and CD44 expression on the surface of PTECs, promoting CaOx crystal adhesion. Notably, pharmacological inhibition of sodium‐glucose cotransporter 2 (SGLT2) can mitigate hyperglycemia‐induced tubular injury, modulate renal glucose metabolism, and reduce kidney stones incidence, highlighting a potential metabolic link between glucose handling and nephrolithiasis [7, 8].

Among the metabolic alterations associated with dysregulated glucose metabolism, lactate has emerged as a critical factor. In diabetic patients, elevated urinary lactate levels are correlated with impaired renal function and increased risk of kidney stone formation [9]. Increased lactate may contribute to stone formation by disrupting renal energy metabolism [10]. In addition to acting as a metabolic end‐product that directly influence stone‐forming processes, lactate can also modify cellular pathways through lactylation [11]. Whether lactate‐driven lactylation participates in CaOx stone pathogenesis remains largely unexplored.

One cellular process that may link metabolic dysregulation to stone formation is epithelial‐mesenchymal transition (EMT). EMT involves the loss of epithelial polarity and cell‐cell junctions, with acquisition of mesenchymal features. Following injury, PTECs may undergo partial EMT, secreting pro‐fibrotic and pro‐inflammatory mediators that promote interstitial fibrosis and tubular dysfunction [12]. These EMT‐related changes can modify the renal microenvironment, enhancing crystal retention and facilitating stone formation [13]. EMT also drives the progression of CKDs, including diabetic nephropathy, where metabolic dysregulation within PTECs triggers phenotypic transitions [2, 14]. However, the involvement of EMT in CaOx stone formation, particularly its metabolic and epigenetic mechanisms have not been fully determined.

In this study, we investigated the role of lactate‐mediated EMT in PTECs exposed to CaOx crystal. We demonstrate that aberrant glucose metabolism promotes SNAIL1 lactylation, which drives EMT and contributes to CaOx stone development. These findings reveal a metabolic–epigenetic axis linking lactate accumulation to tubular phenotypic remodeling and offer new insights into the prevention and treatment of kidney stone disease.

## 2 Results

### 2.1 CaOx Stone Suppresses G6PC and Gluconeogenesis in PTECs

**FIGURE 1:**
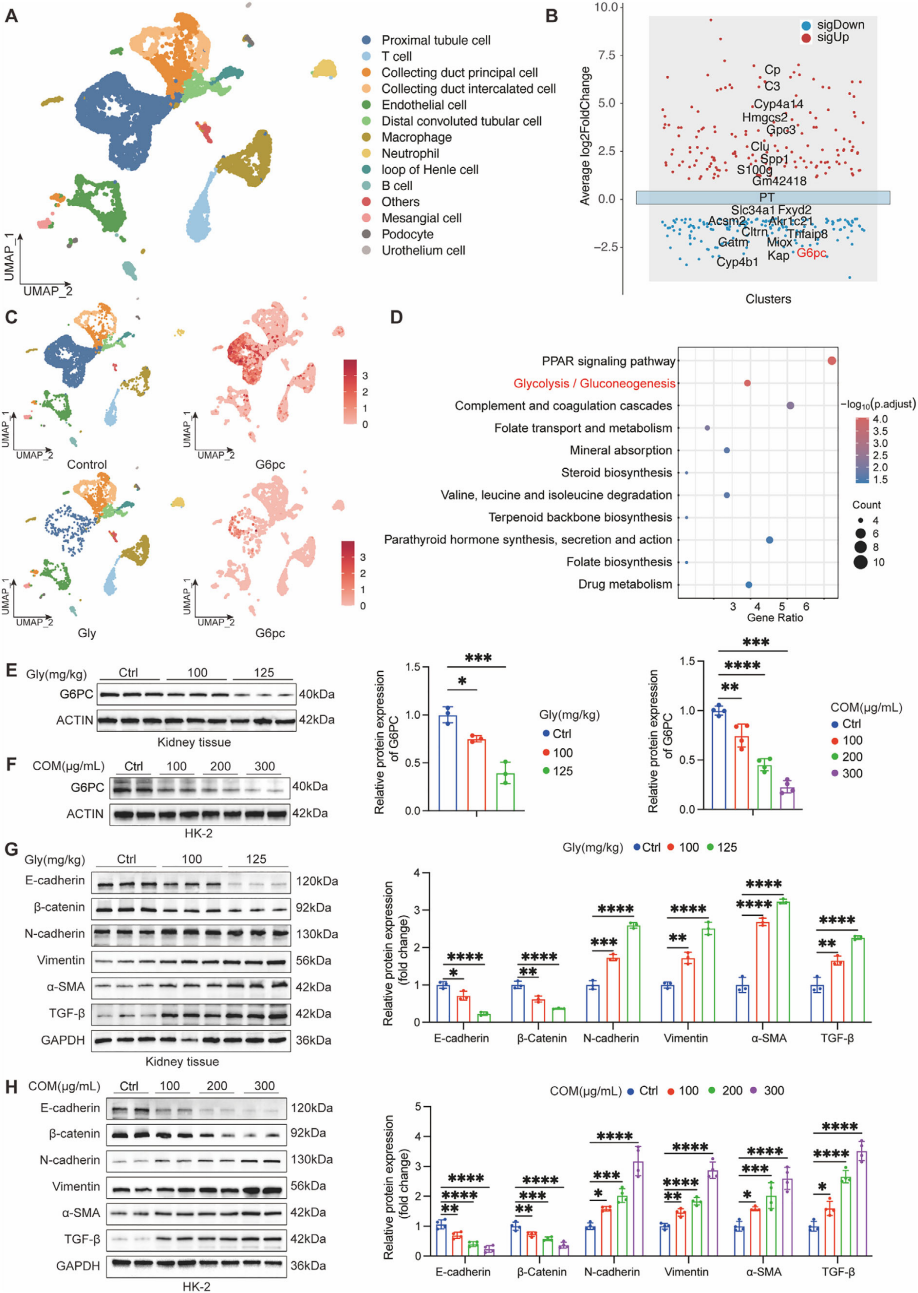
CaOx stone led to G6PC downregulation in tubular epithelium resulting in glucose metabolism dysfunction and EMT. (A) UMAP visualization of 14 distinct cell types in the mouse kidney. (B) Volcano plot of DEGs in mouse renal proximal tubules between the control (Ctrl) and Gly treatment groups. (C) UMAP plots showing cellular distribution across subclusters (left) and G6PC expression (right) in the Ctrl and Gly groups. (D) KEGG pathway enrichment analysis of DEGs in proximal convoluted tubules between the Ctrl and Gly groups. (E) G6PC protein expression in mouse kidney tissues. Representative immunoblots from mice treated with 0 mg/kg (Ctrl), 100 mg/kg, or 125 mg/kg Gly (left) and quantitative analysis (right) (n = 3 per group). (F) G6PC protein expression in HK‐2 cells. Representative immunoblots from cells treated with 0 µg/mL (Ctrl), 100, 200, or 300 µg/mL COM (left) and quantitative analysis (right) (n = 4 per group). (G) Expression of EMT markers in mouse kidney tissues. Representative immunoblots for E‐cadherin, β‐catenin, N‐cadherin, Vimentin, α‐SMA, and TGF‐β (left) and quantitative analysis (right) (n = 3 per group). (H) Expression of EMT markers in HK‐2 cells. Representative immunoblots (left) and quantitative analysis (right) (n = 4 per group). Error bars show the mean ± SD. *p < 0.05, **p < 0.01, ***p < 0.001, ****p < 0.0001. The *p*‐value was determined using one‐way ANOVA (E‐H). All numbers (n) are biologically independent experiments.

Several cell types are involved in the process of kidney stone formation [15]. To determine which cell population is most affected during CaOx stone development, we generated a glyoxylic acid (Gly)‐induced CaOx mouse model and performed single‐cell RNA sequencing (scRNA‐seq) on renal tissues. 14 cell clusters were identified (Figure 1A), and PTECs showed the most pronounced alterations in cell proportion (Figure S1A). As proximal tubule is a major site of filtrate reabsorption and is highly susceptible to CaOx‐induced injury, we focused subsequent transcriptional analyses on PTECs.

Comparative analysis identified differentially expressed genes (DEGs), among which G6PC, a key gluconeogenic enzyme, was significantly altered as shown by Volcano plots (Figure 1B) and UMAP plots (Figure 1C). Furthermore, the downregulation of tubular G6PC was confirmed by spatial transcriptomics of CaOx kidney stone mouse model (Figure S1B). KEGG analysis of the full DEGs revealed marked alterations in glycolysis and gluconeogenesis (Figure 1D). Meanwhile, single‐cell sequencing data of kidneys from clinical cases, showed significant dysregulation of these pathways in proximal tubules, particularly for G6PC (Figure S1C‐G). Furthermore, single‐cell sequencing data revealed that in proximal tubular epithelial cells from both mouse and human kidney stone models, gluconeogenesis‐related genes were downregulated while glycolysis‐related genes were upregulated (Figures S2 and S3). Proteomic and immunohistochemical analyses further confirmed decreased gluconeogenesis and increased glycolysis in the stone‐forming mouse models (Figure S5). Collectively, transcriptional analyses across models established that G6PC was the most downregulated gluconeogenic gene, exhibiting the greatest differential expression in calcium oxalate nephrolithiasis (Figure S4).

Notably, G6PC expression exhibited a dose‐dependent decrease in response to escalating concentrations of Gly in kidney stone models (Figure 1E). This dose‐dependent suppression was also evident in the COM‐treated HK‐2 cell lines (Figure 1F). Consistent with the established literature, Gly or oxalate exposure induced EMT in renal tissues [16] (Figure S7 and S8). EMT also occurred in HK‐2 cells and PTECs following COM treatment (Figure S9). This was evidenced by a marked downregulation of the epithelial junction proteins E‐cadherin and β‐catenin, concomitant with an upregulation of the mesenchymal markers N‐cadherin, Vimentin, α‐SMA, and the pro‐fibrotic cytokine TGF‐β (Figure 1G,H). Collectively, these data demonstrated that the kidney stone milieu was characterized by aberrant glucose metabolism, exemplified by a significant loss of G6PC, and the activation of a pro‐fibrotic EMT program.

### 2.2 G6PC Inhibited Renal Injury and EMT of Tubular Epithelium

**FIGURE 2:**
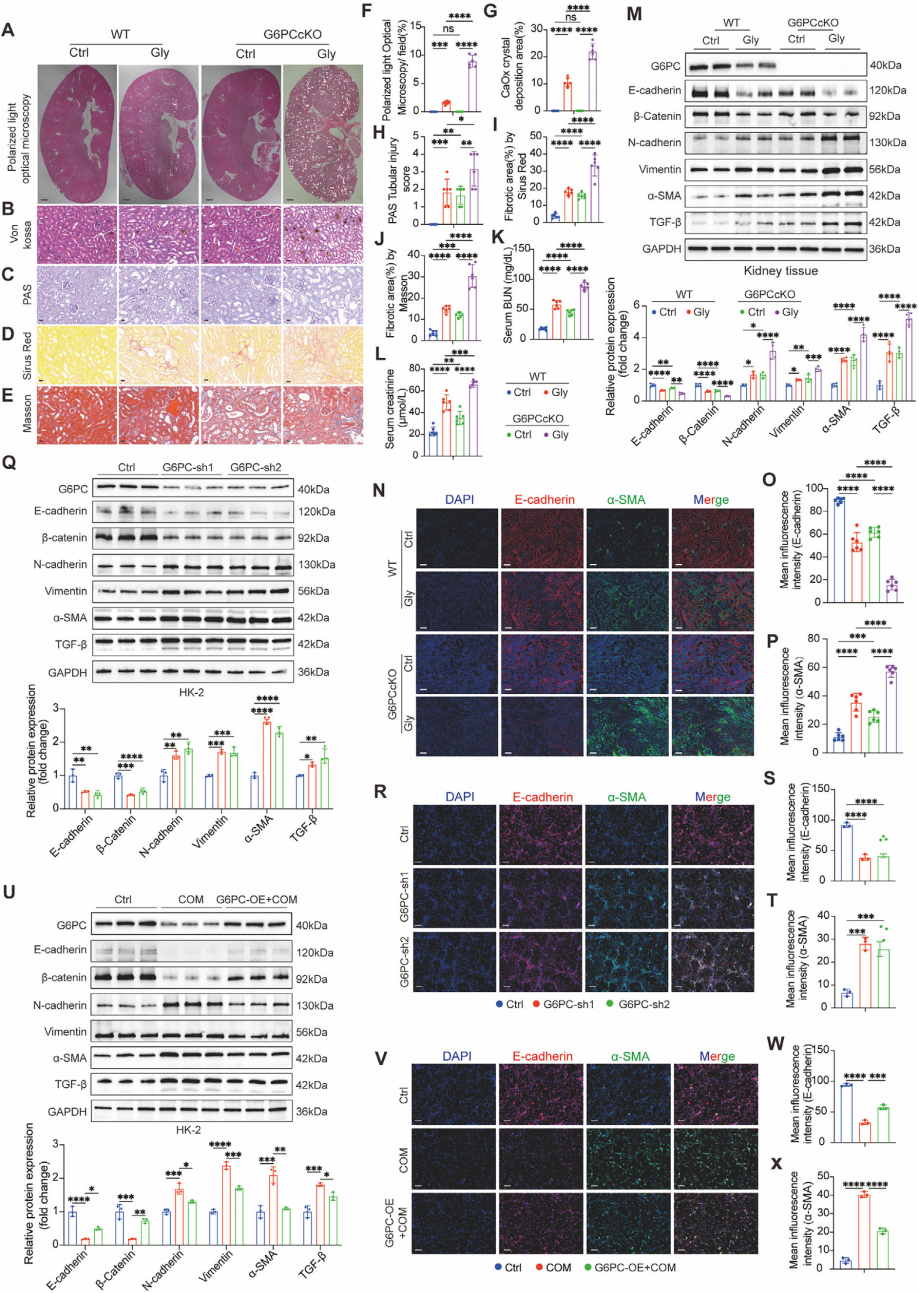
G6PC deficiency promoted renal injury and tubular epithelium EMT in vivo and in vitro. (A‐J) Histological analysis of kidney tissues from WT and G6PCcKO mice following Gly treatment (n = 6 per group for all panels). (A, F) HE staining (scale bar = 500 µm) and quantification of crystal deposition area. (B, G) Von Kossa staining (scale bar = 20 µm) and quantification of CaOx crystal deposition. (C, H) PAS staining (scale bar = 20 µm) and tubular injury score. (D, I) Sirius Red staining (scale bar = 20 µm) and quantification of fibrosis. (E, J) Masson's trichrome staining (scale bar = 20 µm) and quantification of fibrosis. (K, L) Serum levels of blood urea nitrogen (BUN) and creatinine (n = 6 per group). (M) Immunoblotting of EMT‐related markers in kidney tissues from WT and G6PCcKO mice (n = 4 per group). (N‐P) IF of E‐cadherin (red) and α‐SMA (green) in kidney tissues (scale bar = 50 µm, n = 6 per group). (Q) Immunoblotting of EMT‐related markers in control and G6PC‐knockdown (G6PC‐sh) HK‐2 cells (n = 3 per group). (R‐T) IF of E‐cadherin (red) and α‐SMA (green) in HK‐2 cells transfected with G6PC shRNA (scale bar = 50 µm, n = 3 per group). (U) Immunoblotting of EMT‐related markers in control and G6PC‐overexpressing (G6PC‐OE) HK‐2 cells with COM treatment (n = 3 per group). (V‐X) IF of E‐cadherin (red) and α‐SMA (green) in G6PC‐OE HK‐2 cells with COM treatment (scale bar = 50 µm, n = 3 per group). Error bars show the mean ± SD. *p < 0.05, **p < 0.01, ***p < 0.001, ****p < 0.0001. The *p*‐value was determined using one‐way ANOVA (F‐M, O‐Q, S‐U, and W‐X). All numbers (n) are biologically independent experiments.

To confirm the role of G6PC in renal injury by CaOx stone, we crossed Cdh16‐Cre males with G6PC^fl/fl^ females on a C57BL/6J background to generate renal tubular epithelium‐specific G6PC knockout (G6PCcKO) mice (Figure S11). We first assessed the baseline phenotype of G6PCcKO mice. Even in the absence of Gly treatment, G6PCcKO mice exhibited signs of EMT and renal fibrosis compared to wild‐type littermates, as evidenced by histological analyses, immunoblotting, and immunofluorescence. Under Gly treatment, G6PCcKO mice exhibited exacerbated pathological phenotypes compared to the control group. Histological analysis revealed a significant increase in crystal deposition as shown by HE staining (Figure 2A,F) and Von Kossa staining (Figure 2B,G), aggravated tubular injury as shown by PAS staining (Figure 2C,H), and expanded fibrosis as shown by Sirius Red staining (Figure 2D,I) and Masson staining (Figure 2E,J). Consistently, renal function was impaired, as indicated by elevated serum BUN and creatinine levels (Figure 2K,L). Furthermore, immunoblotting demonstrated that G6PC deletion promoted EMT, characterized by decreased epithelial markers and increased interstitial markers in the Gly‐treated group (Figure 2M). Meanwhile, immunofluorescence (IF) showed a decrease of E‐cadherin and an increase of α‐SMA in Gly‐treated G6PCcKO group (Figure 2N). In vitro, knockdown of G6PC in both HK‐2 cells and PTEC cells induced EMT, as demonstrated by immunoblotting and immunofluorescence (Figure 2Q‐T; Figure S13A‐D), whereas overexpression of G6PC attenuated the COM‐induced EMT in these cells (Figure 2U‐X; Figure S1313E‐H). Thus, data confirmed that G6PC exerted protective effects by inhibiting kidney injury, crystal deposition and EMT in the process of kidney stone formation.

### 2.3 Aberrant Glucose Metabolism Leads to Lactate Accumulation and Promotes Tubular EMT

**FIGURE 3:**
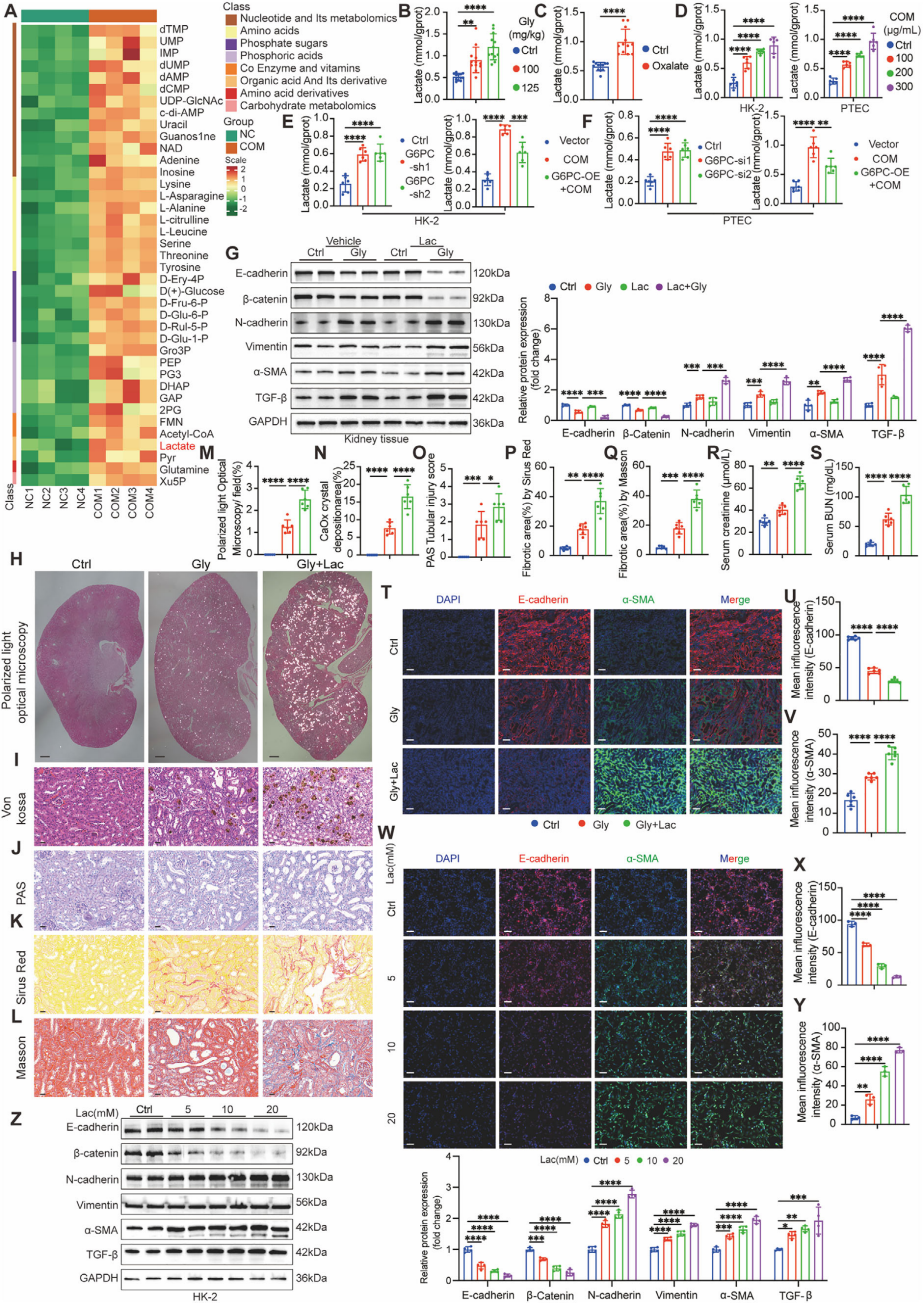
Lactate accumulation drove renal injury and tubular epithelium EMT upon CaOx treatment. (A) Energy metabolomics profiling of altered metabolites in COM‐treated HK‐2 cells (n = 4 per group). (B‐D) Lactate levels in (B, C) kidney tissues from Gly or oxalate‐treated mice (n = 12 per group) and (D) in COM‐treated HK‐2 cells or PTECs (n = 6 per group). (E‐F) Lactate levels in G6PC‐knockdown and G6PC‐overexpressing (E) HK‐2 cell or (F) PTECs (n = 6 per group). (G) Immunoblotting of EMT‐related markers in lactate‐pretreated kidney tissues (n = 4 per group). (H‐Q) Histopathological analysis of lactate‐pretreated kidney tissues (n = 6 per group for all panels). (H, M) HE staining (scale bar = 500 µm) and crystal deposition quantification. (I, N) Von Kossa staining (scale bar = 20 µm) and calcium oxalate crystal quantification. (J, O) PAS staining (scale bar = 20 µm) and tubular injury scoring. (K, P) Sirius Red staining (scale bar = 20 µm) and fibrosis quantification. (L, Q) Masson's staining (scale bar = 20 µm) and fibrosis quantification. (R, S) Serum BUN and creatinine levels in lactate‐pretreated kidney tissues (n = 6 per group). (T‐V) Immunofluorescence of E‐cadherin (red) and α‐SMA (green) in lactate‐pretreated kidney tissues (scale bar = 50 µm, n = 6 per group). (W‐Y) Immunofluorescence of E‐cadherin (red) and α‐SMA (green) in lactate‐treated HK‐2 cells (scale bar = 50 µm, n = 3 per group). (Z) Immunoblotting of EMT‐related markers in lactate‐treated HK‐2 cells (n = 3 per group). Error bars show the mean ± SD. *p < 0.05, **p < 0.01, ***p < 0.001, ****p < 0.0001. The *p*‐value was determined using two‐tailed unpaired Student's test (C) or one‐way ANOVA (B, D‐G, M‐S, U‐V, and X‐Z). All numbers (n) are biologically independent experiments.

G6PC plays a critical role in glucose metabolism. To investigate how aberrant glucose metabolism contributes to CaOx‐induced renal injury, the energy metabolome of four control HK‐2 samples and four COM‐treated HK‐2 cells was profiled. Metabolomics revealed a significant accumulation of lactate, a gluconeogenic precursors, in COM‐treated cells (Figure 3A). Simultaneously, levels of other key gluconeogenic/glycolytic intermediates, including glucose‐6‐phosphate, fructose‐6‐phosphate, and pyruvate, were also elevated, painting a comprehensive picture of impaired gluconeogenic flux and diverted glycolytic metabolism (Figure 3A; Figure S6). This finding aligned with the observed downregulation of G6PC, the terminal enzyme of gluconeogenesis. A dose‐dependent increase in lactate levels was observed in the kidneys of mice treated with either Gly or oxalate (Figure 3B,C).  Furthermore, in vitro experiments demonstrated that lactate levels increased with COM treatment in a dose dependent manner in HK‐2 cells and primary PTECs (Figure 3D). Consistent with impaired G6PC expression, knockdown of G6PC resulted in lactate accumulation in HK‐2 cells and PTECs (Figure 3E,F). Conversely, overexpression of G6PC attenuated the COM‐induced elevation in lactate levels (Figure 3E,F). Administration of exogenous lactate markedly aggravated renal injury in kidney stone models (Figure 3H,M). This was evidenced by increased crystal deposition as shown by Von Kossa staining (Figure 3I,N), more severe tubular injury as shown by PAS staining (Figure 3J,O), enhanced fibrosis as shown by Sirius Red staining (Figure 3K,P) and Masson's staining (Figure 3L,Q), and ultimately, impaired renal function (Figure 3R,S). Moreover, immunoblotting and immunofluorescence analyses confirmed that lactate promoted EMT in vivo in kidney stone models (Figure 3G,T‐V). Consistently, in cultured HK‐2 cells and primary PTECs, lactate induced EMT in a dose‐dependent manner, as shown by immunoblotting and immunofluorescence (Figure 3W‐Z; Figure S14). Together, these data suggest that lactate accumulation contributes to the induction of EMT both in vivo and in vitro.

### 2.4 G6PC Downregulation Promoted EMT of Tubular Epithelium Through the TGF‐β/SMAD3 Pathway Mediated by SNAIL1

**FIGURE 4:**
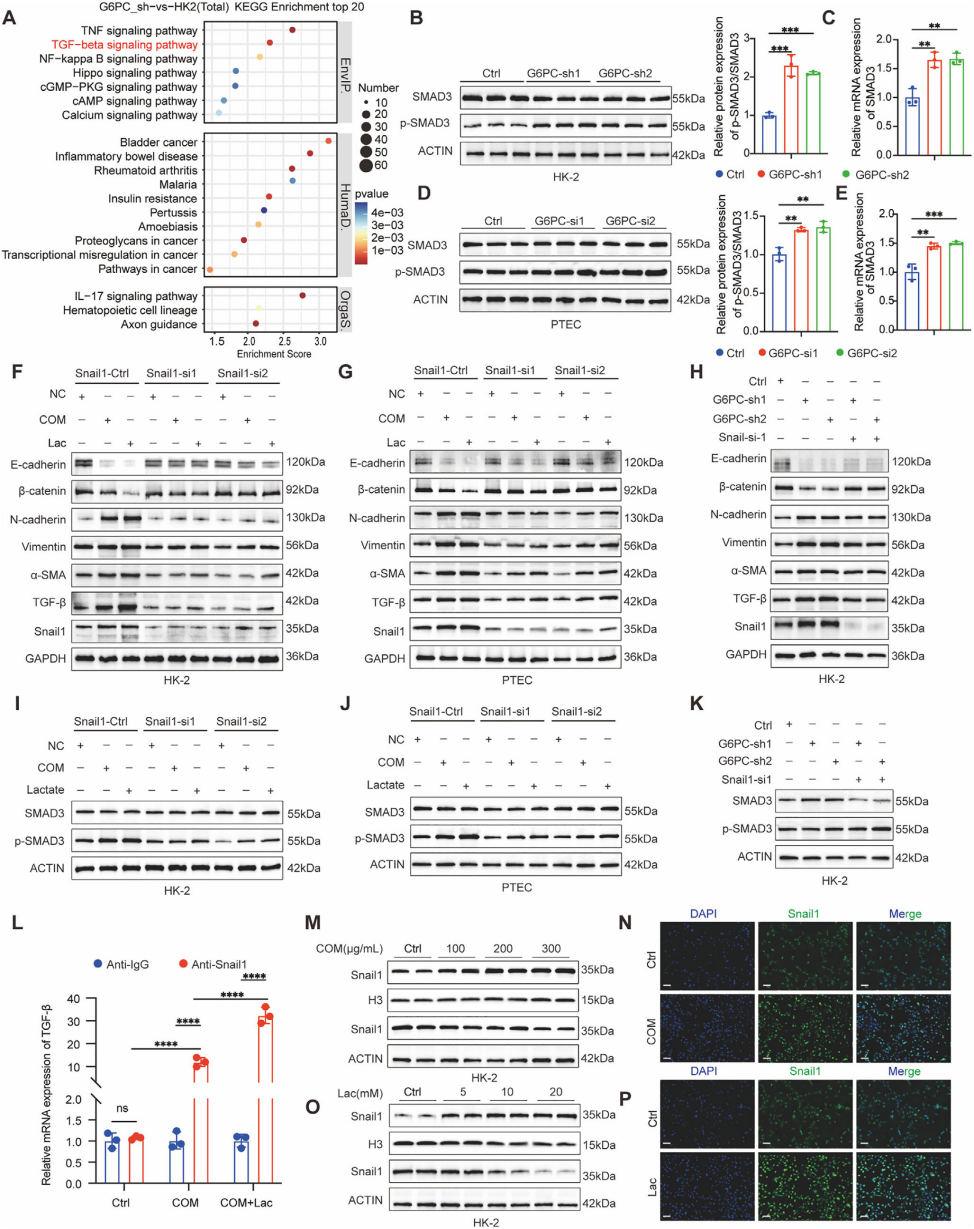
SNAIL1 mediated EMT of tubular epithelium through TGF‐β/SMAD3 pathway. (A) Top 20 significantly altered KEGG pathways in G6PC‐knockdown HK‐2 cells. (B, D) Immunoblotting (left) and quantification (right) of SMAD3 phosphorylation in G6PC‐knockdown HK‐2 cells or PTECs (n = 3 per group). (C, E) The relative mRNA levels of SMAD3 in G6PC knockdown HK‐2 cells or PTECs (n = 3 per group). (F‐H) Immunoblotting of EMT‐related markers in (F, G) SNAIL1‐knockdown HK‐2 cells or PTECs treated with COM or lactate; (H) HK‐2 cells with concomitant SNAIL1 and G6PC knockdown (n = 3 per group). (I‐K) Immunoblotting of SMAD3 phosphorylation in (I, J) SNAIL1‐knockdown HK‐2 cells or PTECs treated with COM or lactate; (K) HK‐2 cells with concomitant SNAIL1 and G6PC knockdown (n = 3 per group). (L) The relative mRNA levels of TGF‐β binding to anti‐SNAIL1 antibody in HK‐2 cells treated with COM and lactate in CHIP assay (n = 3 per group). (M, O) Immunoblotting of SNAIL1 in cytoplasmic and nuclear fractions of HK‐2 cells treated with increasing concentrations of (M) COM or (O) lactate (n = 4 per group). (N, P) Immunofluorescence showing SNAIL1 (green) localization in HK‐2 cells treated with (N) COM or (P) lactate. Nuclei are stained with DAPI (blue) (scale bar = 50 µm, n = 3 per group). Error bars show the mean ± SD. *p < 0.05, **p < 0.01, ***p < 0.001, ****p < 0.0001. The *p*‐value was determined using one‐way ANOVA (B‐E and L). All numbers (n) are biologically independent experiments.

To investigate the molecular mechanisms linking G6PC depletion to EMT, RNA sequencing in G6PC‐knockdown HK‐2 cells was performed. KEGG pathway analysis identified the TGF‐β signaling pathway as one of the most significantly altered pathways (Figure 4A). Consistent with this result, G6PC depletion increased TGF‐β pathway activity in both HK‐2 cells and primary cultured PTECs, as evidenced by elevated SMAD3 mRNA expression (Figure 4C,E) and enhanced SMAD3 phosphorylation (Figure 4B,D). Notably, phosphorylation of SMAD3, but not SMAD2, was selectively increased during calcium oxalate crystal exposure (Figure S15A–L), indicating preferential activation of SMAD3 signaling (Figure S15A‐L). Similarly, increased SMAD3 phosphorylation was observed in kidneys from G6PC conditional knockout mice (Figure S15 M). In addition, lactate treatment also activated TGF‐β/SMAD3 signaling in HK‐2 cells and primary PTECs (Figure S16), linking metabolic alterations to pathway activation.

To identify the upstream regulator connecting G6PC deficiency to TGF‐β pathway activation, we performed a targeted screen of transcription factors including SNAIL1, SNAIL2, TWIST1, and ZEB1 implicated in EMT. This screen identified SNAIL1 as a candidate mediator (Figure S17). Functional validation demonstrated that SNAIL1 knockdown reversed COM‐ or lactate‐induced EMT in HK‐2 cells and primary PTECs (Figure 4F,G). Furthermore, in G6PC‐knockdown cells, additional SNAIL1 silencing further elevated epithelial markers while suppressing mesenchymal markers, accompanied by suppression of TGF‐β signaling (Figure 4H), positioning SNAIL1 as a critical downstream effector of G6PC. Mechanistically, SNAIL1 knockdown attenuated COM‐ or lactate‐induced SMAD3 phosphorylation in HK‐2 cells and primary PTECs (Figure 4I,J). It is well established that the transcription factor SNAIL1 promotes the EMT process [17]. More importantly, our chromatin immunoprecipitation (ChIP) assays further demonstrated the direct binding of SNAIL1 to the TGF‐β promoter, supporting its role in transcriptional activation of TGF‐β signaling (Figure 4L). Consistently, lactate or COM treatment induced dose‐dependent nuclear translocation of SNAIL1 in HK‐2 cells (Figure 4M,O), as confirmed by subcellular fractionation (Figure 4M,O), and immunofluorescence staining visually corroborated this localization shift (Figure 4N,P). Together, these data indicate G6PC downregulation promotes tubular EMT through activation of a SNAIL1‐dependent TGF‐β/SMAD3 signaling pathway in the context of kidney stone formation.

### 2.5 G6PC Downregulation Induced CBP/p300‐Mediated Lactylation of SNAIL1

**FIGURE 5:**
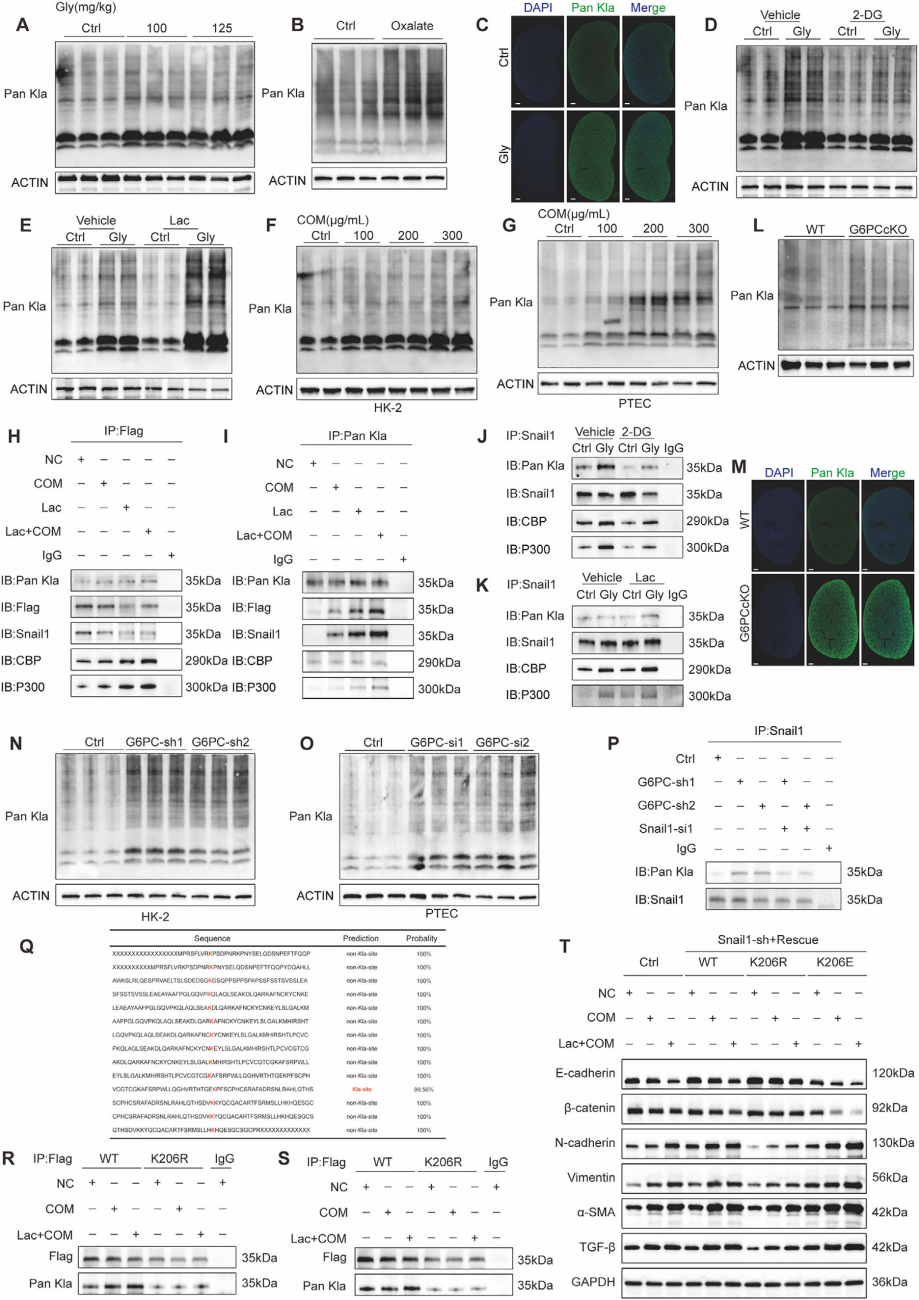
G6PC deficiency promoted CBP/p300‐mediated lactylation of SNAIL1 at lysine 206. (A‐C) Immunoblotting (A, B) and immunofluorescence (C) of pan‐lysine lactylation (Pan‐Kla) levels in kidney tissues from mice treated with Gly or oxalate (n = 3–6 per group). (D‐G) Immunoblotting of Pan‐Kla levels in (D, E) mouse kidney tissues pretreated with 2‐DG or lactate and (F, G) COM‐treated HK‐2 cells or PTECs (n = 4 per group). (H‐K) Co‐immunoprecipitation of SNAIL1 lactylation and its interaction with CBP/p300 in HK‐2 cells under various treatments (n = 3 per group). (H‐I) Cells were treated with COM or lactate. (J‐K) Cells were pretreated with 2‐DG or lactate prior to Gly treatment. Immunoprecipitation was performed using (H) anti‐Flag, (I) anti‐pan‐Kla, or (J, K) anti‐SNAIL1 antibodies. (L‐O) Pan‐Kla levels were examined in vivo and in vitro (n = 3 per group). (L, M) Levels in kidneys of G6PCcKO mice were assessed by (L) immunoblotting and (M) immunofluorescence. (N, O) Levels in G6PC‐knockdown (N) HK‐2 or (O) PTEC cells were assessed by immunoblotting. (P) Immunoblotting of SNAIL1 lactylation in concomitant G6PC and SNAIL1 knockdown HK‐2 cells (n = 3 per group). (Q) Prediction of the SNAIL1 lactylation site at lysine 206 (K206) using the DeepKlapred database. (R, S) Immunoblotting of lactylation levels of wild‐type (WT) and K206R mutant SNAIL1 in COM/lactate‐treated HK‐2 cells (R) or PTECs (S) (n = 3 per group). (T) Immunoblotting of EMT‐related markers in SNAIL1‐knockdown HK‐2 cells reconstituted with WT, K206R, or K206E SNAIL1 mutants under COM/lactate treatment (n = 3 per group). All numbers (n) are biologically independent experiments.

Lactate is a known regulator of protein lactylation. However, its role and alteration in kidney stone models had not been fully elucidated. The kidney stone models exhibited a lactate dose‐dependent increase in lactylation levels, as determined by immunoblotting and immunofluorescence (Figure 5A‐C). In vivo, lactylation levels were reduced by treatment with the glycolysis inhibitor 2‐deoxy‐D‐glucose (2‐DG) (Figure 5D), and were markedly increased following exogenous lactate administration (Figure 5E). Similarly, COM treatment induced a dose‐dependent increase in protein lactylation in HK‐2 cells and primary PTECs (Figure 5F,G).

To determine whether SNAIL1 undergoes lactylation under these conditions, HK‐2 cells treated with COM, lactate, or their combination, SNAIL1 lactylation was consistently enhanced. Notably, we observed increased SNAIL1 lactylation accompanied by a reduction in total SNAIL1 protein levels (Figure 5H). Consistently, IP with a pan‐lysine lactylation (Kla) antibody confirmed the elevated level of the lactylated SNAIL1 (Figure 5I). In contrast, inhibition of glycolysis by 2‐DG reduced SNAIL1 lactylation (Figure 5J), whereas lactate supplementation increased it in renal stone cell lines as demonstrated by IP with an anti‐SNAIL1 antibody (Figure 5K).

CBP/p300, a well‐characterized acetyltransferase previously implicated in SNAIL1 regulation, has also been reported to mediate protein lactylation [18]. We performed co‐immunoprecipitation (Co‐IP) to confirm a physical interaction between SNAIL1 and CBP/p300 (Figure 5H‐K). Notably, CBP/p300 protein levels were positively regulated by lactate, increasing upon lactate supplementation and decreasing following lactate depletion (Figure 5H‐K). Consistent with these findings, global protein lactylation was elevated in kidneys from G6PC conditional knockout mice (Figure 5L,M) as well as in HK‐2 cells and primary PTECs following G6PC knockdown (Figure 5N,O). Importantly, knockdown of SNAIL1 selectively reduced the lactylated protein signal, indicating that SNAIL1 represents a major lactylated target under these conditions (Figure 5P).

Next, to identify the specific lactylation site on SNAIL1, lysine 206 (K206) was predicted as a candidate residue using the DeepKlaPred database (Figure 5Q). To validate this prediction, site‐directed mutants of SNAIL1 were generated and expressed in HK‐2 cells and primary PTECs. Mutation of K206 to arginine (K206R) markedly reduced SNAIL1 lactylation, supporting K206 as a critical lactylation site (Figure 5R,S). In contrast, mutation of K206 to glutamic acid (K206E), which mimics constitutive modification, exacerbated EMT even in the context of SNAIL1 knockdown (Figure 5T). Together, these results indicate that G6PC downregulation enhances CBP/p300‐mediated lactylation of SNAIL1 at the functionally important K206 residue, thereby promoting EMT during kidney stone formation.

### 2.6 Inhibition of TGF‐β/SMAD3 Signaling Attenuates COM‐Induced EMT and Renal Fibrosis

**FIGURE 6:**
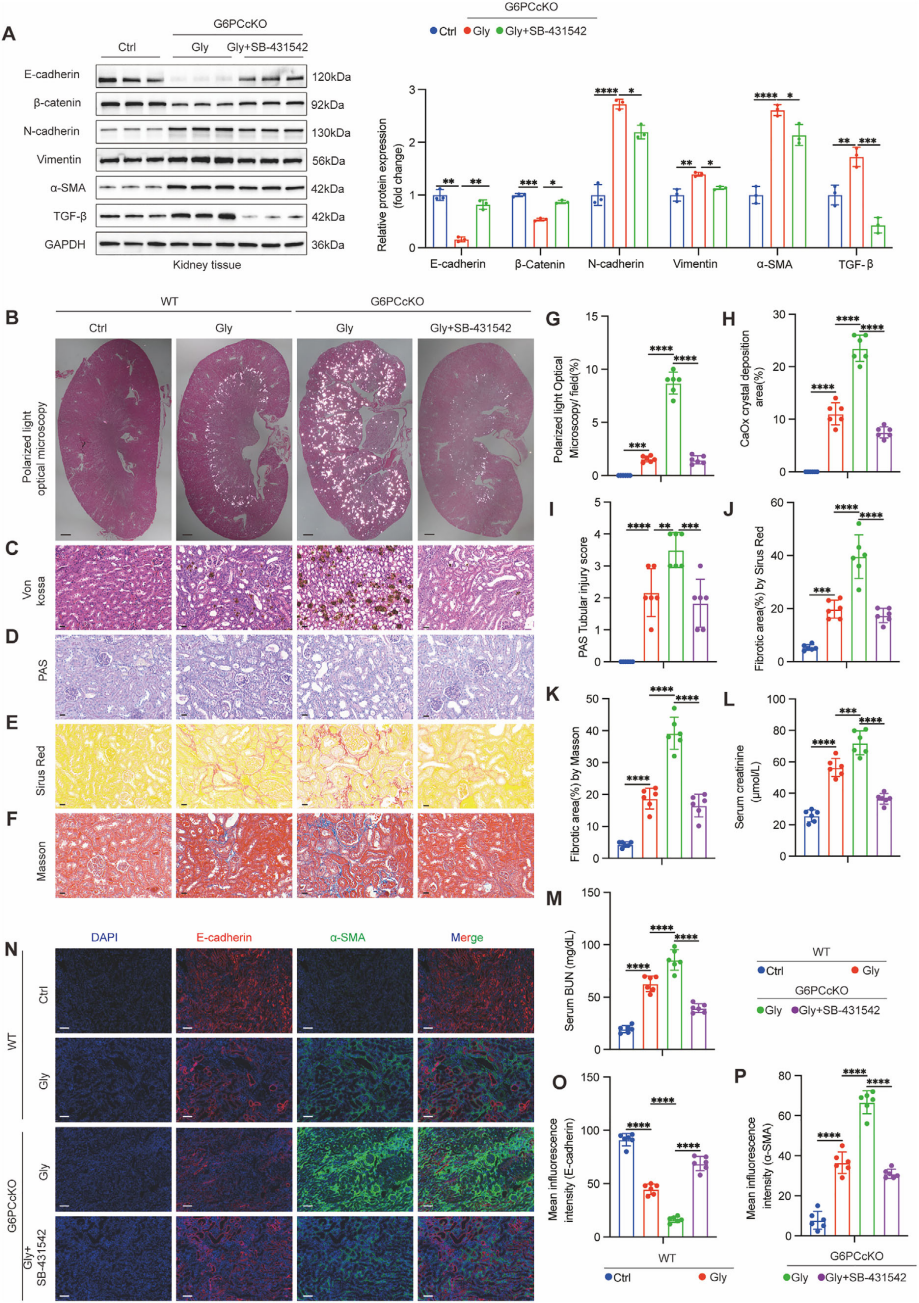
Therapeutic inhibition of TGF‐β/Smad3 signaling ameliorated COM‐induced EMT and renal fibrosis. (A) Immunoblotting and quantification of EMT‐related markers in kidney tissues from G6PCcKO mice treated with Gly and SB‐431542 (n = 3 per group). (B‐K) Histopathological analysis of kidney tissues from G6PCcKO mice treated with Gly and SB‐431542 (n = 6 per group for all panels). (B, G) HE staining (scale bar = 500 µm) and quantification of crystal deposition area. (C, H) Von Kossa staining (scale bar = 20 µm) and quantification of calcium oxalate deposition. (D, I) PAS staining (scale bar = 20 µm) and tubular injury scoring. (E, J) Sirius Red staining (scale bar = 20 µm) and quantification of fibrotic area. (F, K) Masson's trichrome staining (scale bar = 20 µm) and quantification of fibrotic area. (L, M) Serum BUN and creatinine levels in G6PCcKO mice treated with Gly and SB‐431542 (n = 6 per group). (N‐P) Immunofluorescence and quantification of E‐cadherin (red) and α‐SMA (green) in kidney tissues from WT and G6PCcKO mice treated with Gly and SB‐431542 (scale bar = 50 µm, n = 6 per group). Error bars show the mean ± SD. *p < 0.05, **p < 0.01, ***p < 0.001, ****p < 0.0001. The *p*‐value was determined using one‐way ANOVA (A, G‐M, and O‐P). All numbers (n) are biologically independent experiments.

Given that activation of the TGF‐β/SMAD3 pathway is closely associated with EMT, the TGF‐β inhibitor SB‐431542 was administered to G6PC conditional knockout mice. SB‐431542 treatment significantly alleviated EMT (Figure 6A) and attenuated renal pathology in vivo (Figure 6B,G). Histological analyses revealed reduced crystal deposition (Figure 6C,H), diminished tubular injury (Figure 6D,I), and decreased interstitial fibrosis, as demonstrated by Sirius Red and Masson's trichrome staining (Figure 6E–F,J–K). Consistent with these pathological improvements, serum creatinine (Figure 6L) and BUN levels (Figure 6M) were significantly reduced following SB‐431542 treatment. The amelioration of EMT was further confirmed by immunofluorescence analysis of epithelial and mesenchymal markers (Figure 6N‐P). Collectively, these data indicate that pharmacological inhibition of TGF‐β signaling with SB‐431542 mitigates COM‐induced EMT and renal fibrosis, thereby counteracting the deleterious effects of G6PC deficiency in PTECs.

### 2.7 Reduction of Lactate Levels Mitigated COM‐Induced EMT and Renal Fibrosis

**FIGURE 7:**
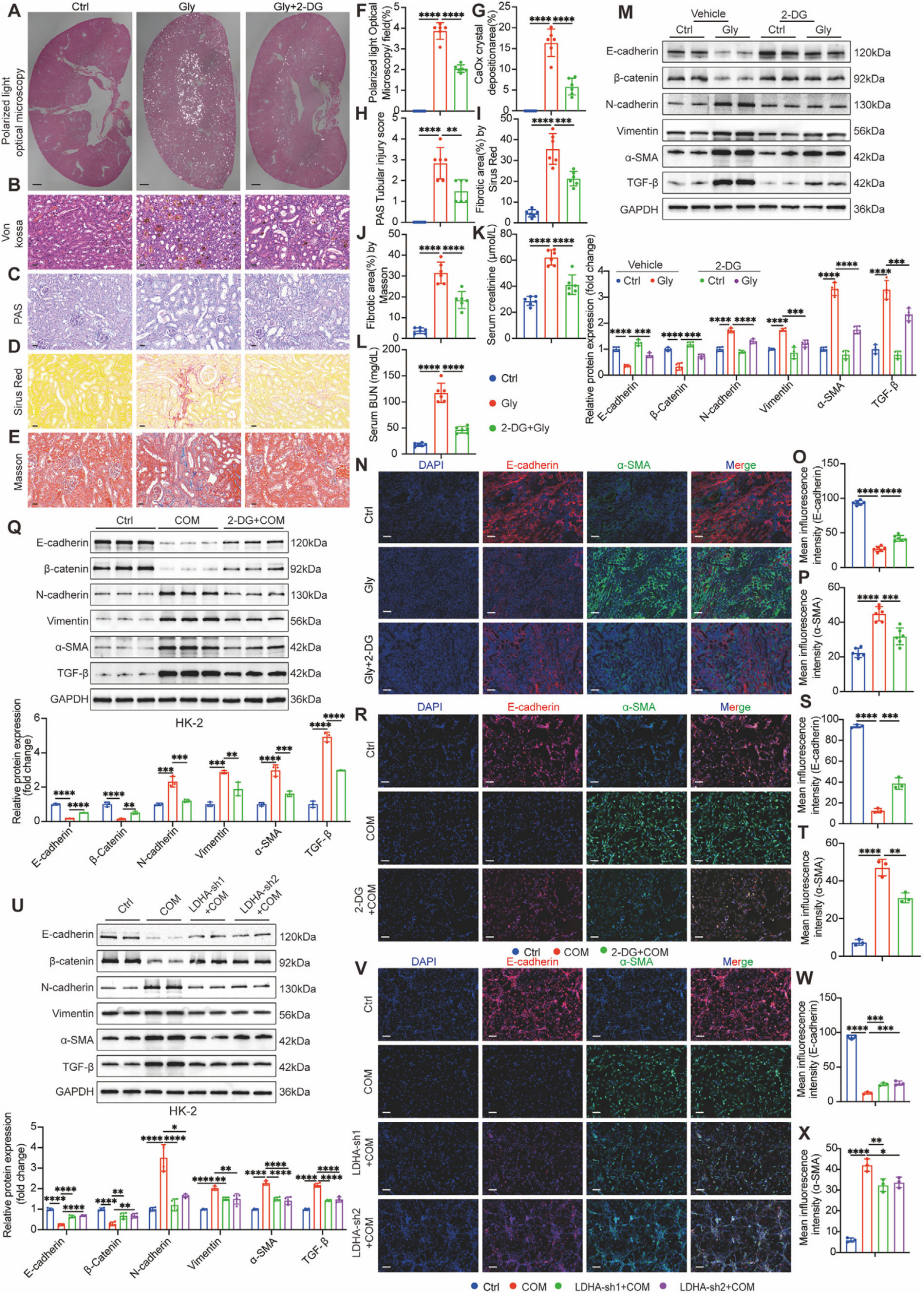
Metabolic intervention by lactate reduction attenuated COM‐induced renal fibrosis and EMT. (A‐J) Histopathological analysis of kidney tissues from mice treated with Gly with or without 2‐DG (n = 6 per group for all panels). (A, F) HE staining (scale bar = 500 µm) and quantification of crystal deposition area. (B, G) Von Kossa staining (scale bar = 20 µm) and quantification of calcium oxalate deposition. (C, H) PAS staining (scale bar = 20 µm) and tubular injury scoring. (D, I) Sirius Red staining (scale bar = 20 µm) and quantification of fibrotic area. (E, J) Masson's trichrome staining (scale bar = 20 µm) and quantification of fibrotic area. (K, L) Serum BUN and creatinine levels in mice treated with Gly with or without 2‐DG (n = 6 per group). (M) Immunoblotting and quantification of EMT‐related markers in kidney tissues from mice treated with Gly with or without 2‐DG (n = 6 per group). (N‐P) Immunofluorescence and quantification of E‐cadherin (red) and α‐SMA (green) in kidney tissues from mice treated with Gly with or without 2‐DG (scale bar = 50 µm, n = 6 per group). (Q) Immunoblotting and quantification of EMT‐related markers in HK‐2 cells treated with COM with or without 2‐DG (n = 6 per group). (R‐T) Immunofluorescence and quantification of E‐cadherin (red) and α‐SMA (green) in HK‐2 cells treated with COM with or without 2‐DG (scale bar = 50 µm, n = 6 per group). (U) Immunoblotting and quantification of EMT‐related markers in LDHA‐knockdown HK‐2 cells treated with COM (n = 6 per group). (V‐X) Immunofluorescence and quantification of E‐cadherin (red) and α‐SMA (green) in LDHA‐knockdown HK‐2 cells treated with COM (scale bar = 50 µm, n = 6 per group). Error bars show the mean ± SD. *p < 0.05, **p < 0.01, ***p < 0.001, ****p < 0.0001. The *p*‐value was determined using one‐way ANOVA (F‐M, O‐Q, S‐U, and W‐X). All numbers (n) are biologically independent experiments.

To assess the contribution of lactate to kidney stone‐associated renal injury, the glycolytic inhibitor 2‐DG was used to reduce endogenous lactate production in vivo. Pre‐treatment with 2‐DG attenuated kidney injury in mice, as evidenced by reduced crystal deposition (Figure 7B,G), mitigated tubular injury (Figure 7C,H), and decreased renal fibrosis (Figure 7D‐E,I,J) in kidney stone models. Consistent with these pathological improvements, renal function was preserved, as indicated by lower serum creatinine (Figure 7K) and BUN levels (Figure 7L).

The attenuation of renal injury was accompanied by a reduction in EMT, as confirmed by immunoblotting (Figure 7M) and immunofluorescence (Figure 7N‐P). Consistent with the in vivo findings, 2‐DG pre‐treatment similarly suppressed EMT in HK‐2 cells by immunoblotting (Figure 7Q) and immunofluorescence (Figure 7R‐T). To further validate the role of lactate in mediating EMT, lactate dehydrogenase A (LDHA) was knocked down to reduce lactate production in HK‐2 cells. LDHA silencing significantly attenuated COM‐induced EMT compared with COM treatment alone (Figure 7U‐X; Figure S19). Thus, our study established that reducing lactate production mitigated COM‐induced EMT in tubular epithelium and subsequent renal fibrosis.

## 3 Discussion

EMT in renal tubular epithelial cells predisposes the kidney to crystal deposition and adhesion, a critical early step in stone formation [9]. EMT contributes to nephrolithiasis through two mechanisms: first, by directly promoting crystal adhesion and aggregation [19]; and second, by generating myofibroblasts that secrete fibronectin, osteopontin, and pro‐inflammatory factors. These processes accelerate extracellular matrix accumulation and amplify inflammatory responses, thereby establishing a self‐reinforcing “stone–fibrosis” cycle [20]. In parallel, numerous clinical studies have underscored a close association between disturbances in glucose metabolism and the risk of nephrolithiasis [21, 22]. Given the high energy demand of PTECs and their susceptibility to metabolic reprogramming under pathological conditions, understanding how glucose metabolism contributes to stone pathogenesis is of particular importance.

Using a mouse kidney stone model, we identified a significant downregulation of G6PC, a key enzyme in renal gluconeogenesis. G6PC dysregulation initiates a pathogenic cascade in the kidney. In hereditary G6PC deficiency, such as glycogen storage disease type I, renal involvement manifests as Fanconi syndrome and metabolic abnormalities that can progress to nephrocalcinosis and CKD. In acquired conditions, including diabetic nephropathy, hyperglycemia‐induced G6PC suppression leads to G6P accumulation, endoplasmic reticulum stress, and apoptosis, which ultimately contributing to proteinuria and fibrosis [23]. By manipulating G6PC expression and employing energy metabolomics, we demonstrate that G6PC suppression in the context of stone formation is accompanied by impaired gluconeogenesis and a concomitant accumulation of lactate.

Lactate has been increasingly recognized as an active metabolic signal in kidney disease rather than a passive by‐product of glycolysis. While previous research has established the role of lactate in renal diseases, such as diabetic nephropathy [24], our study reveals that modulating lactate levels, either by targeting LDHA or pharmacologically, can influence stone formation. Lactate levels, in turn, influence the global protein lactylation landscape. Lysine lactylation, a metabolite‐derived post‐translational modification, is dynamically regulated by lactate homeostasis and competes with other modifications like acetylation and methylation for shared residues on histones. The functional relevance of lactylation has been documented across diverse pathological contexts, including cancer [25], neurodegeneration [26], myocardial injury [27, 28], and kidney disease. In renal fibrosis, PKM2‐driven lactate overproduction in tubular cells promotes histone lactylation, leading to upregulation of TGF‐β1 and subsequent activation of Smad3 signaling in macrophages [29, 30]. Additionally, lactylation of histone H3 at lysine 18 (H3K18la) and of the protein Ezrin has been implicated in sepsis‐associated acute kidney injury [31]. Separately, HSPA12A facilitates renal repair after ischemia/reperfusion injury by enhancing c‐Myc lactylation, which promotes the proliferation of proximal tubular epithelial cells [32]. Despite these advances, the role of lactylation in nephrolithiasis has remained largely unexplored.

TGF‐β signaling is a master regulator of EMT in the kidney, with SMAD3 serving as its primary downstream effector. The expression of TGF‐β itself is regulated by SNAIL1, a key transcription factor that initiates EMT. Nuclear translocation of SNAIL1 is essential for TGF‐β transcription and subsequent activation of the TGF‐β/SMAD3 pathway [33]. Post‐translational modifications including methylation, acetylation, and ubiquitination control SNAIL1 nuclear import, with acetylation inhibition promoting its translocation [34, 35]. These observations led us to hypothesize that acetylation and lactylation might act antagonistically. We speculated that CBP/p300, an acetyltransferase known to interact with SNAIL1, could also function as a lactyltransferase to modify lysine residues. Our Co‐IP experiments showing enhanced SNAIL1‐CBP/p300 interaction following lactate treatment support this idea. However, the precise mechanism underlying this antagonism remains to be determined.

We found increased lactylation at K206 of SNAIL1, which facilitates its nuclear translocation and enhances TGF‐β transcription. Using site‐directed mutagenesis, we validated that lactylation at K206 is critical for SNAIL1‐mediated regulation of EMT‐related pathways. This finding aligns with the work in lung cancer showing that targeting SNAIL1 can reverse EMT [36]. We therefore propose that modulating SNAIL1 lactylation represents a potential therapeutic avenue for kidney stones.

**FIGURE 8:**
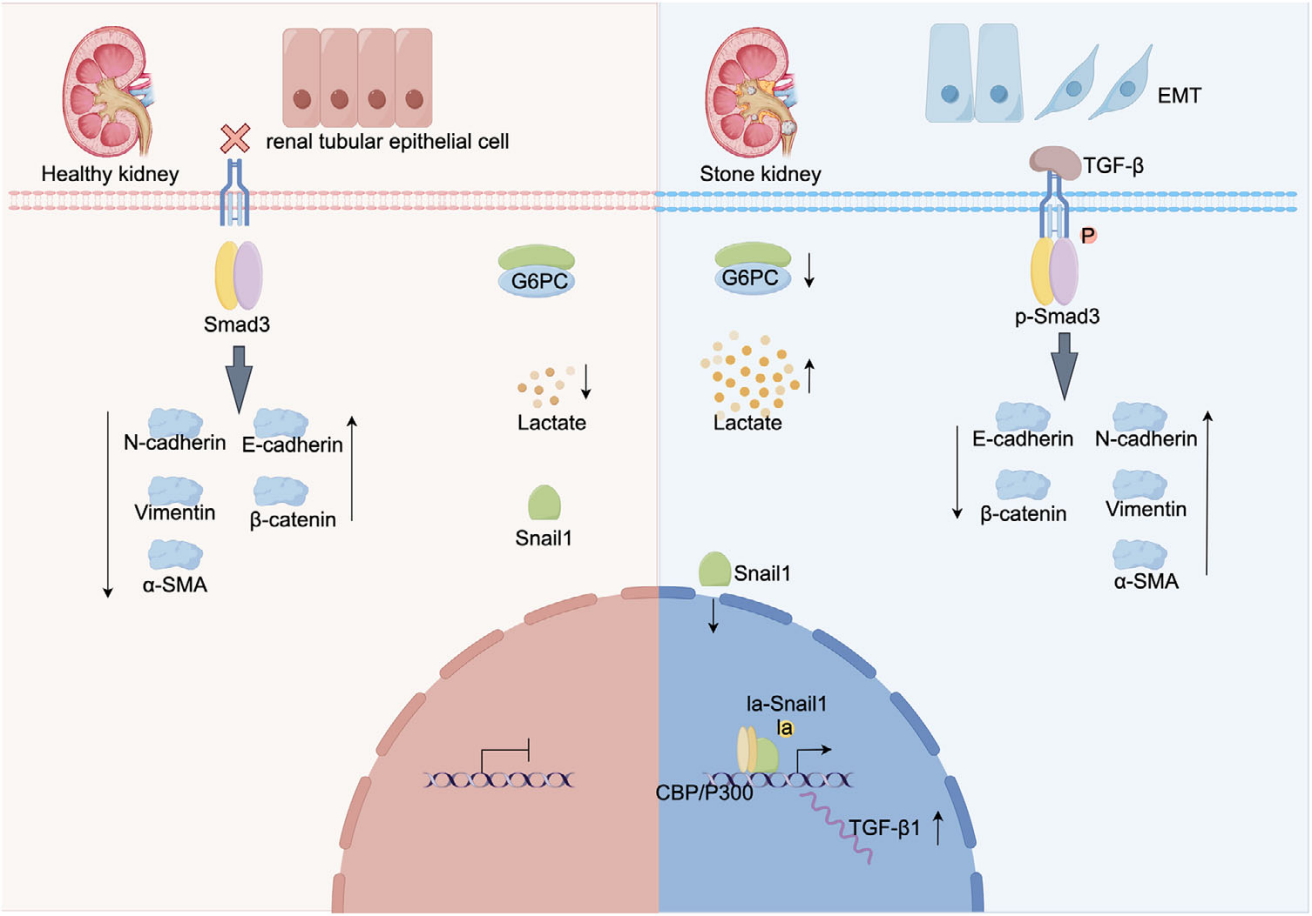
Schematic diagram of SNAIL1 K206 lactylation‐dependent TGF‐β/SMAD3 activation mediated G6PC deficiency‐induced renal CaOx Stone injury. Upon CaOx treatment, impaired renal PTECs exhibited reduced G6PC levels and consequent lactate accumulation. This metabolic shift promoted the CBP/p300‐mediated lactylation of SNAIL1 at the K206 residue. This modification drove the nuclear translocation of SNAIL1, which subsequently upregulated TGF‐β expression and activated the TGF‐β/SMAD3 signaling pathway. Ultimately, this cascade enhanced the EMT in renal PTECs and accelerated renal fibrosis, thereby facilitating the deposition of calcium oxalate crystals and the progression of stone formation.

In summary, our study delineated a novel metabolic‐epigenetic axis in calcium oxalate stone formation. Metabolic dysregulation in tubular epithelium causes transcriptional downregulation of G6PC, leading to lactate accumulation. Elevated lactate promotes CBP/p300‐mediated lactylation of SNAIL1 at K206, facilitating its nuclear translocation. Nuclear SNAIL1 upregulates TGF‐β expression, activating TGF‐β/SMAD3 signaling. This cascade drives EMT and renal fibrosis in PTECs, establishing a microenvironment that accelerates calcium oxalate crystal deposition and the pathogenesis of kidney stone formation (Figure 8).

## 4 Experimental Section

### 4.1 Single Cell Sequencing and Data Analysis

We performed single‐cell RNA sequencing to analyze renal cells from mice in the control and glyoxylic acid‐induced modeling groups, which were dissociated into single‐cell suspensions according to the protocol outlined in our previous work [37]. Meanwhile, we also analyzed single‐cell RNA sequencing results of Randall's plaques from kidney stone patients and normal renal papillary tissues from healthy individuals in the GEO public database (GSE176155). For quality control, cells were retained if they met the following criteria: genes detected per cell between 200 and 8000, UMIs per cell greater than 400, and a mitochondrial UMI ratio below 50%. All downstream analyses were conducted using the “Seurat” package in R. Highly variable genes were identified with the “FindVariableFeatures” function and used for principal component analysis (PCA) and unsupervised clustering. Cell clusters were annotated by determining cluster‐specific marker genes via the “FindAllMarkers” function, followed by cross‐referencing with the CellMarker database and established literature markers. Finally, KEGG pathway enrichment analysis was performed on differentially expressed genes.

### 4.2 Spatial Transcriptomic Analysis

Spatial transcriptomics analysis was carried out on kidney tissue sections from a glyoxylic acid–induced nephrolithiasis C57BL/6 mouse and an age‐matched untreated control, utilizing the 10x Genomics Visium platform. Raw count matrices were processed in R with Seurat (version 4.4.0). Data normalization and variance stabilization were performed using SCTransform under default settings. Principal component analysis (PCA) was applied to the scaled expression matrix. The number of principal components retained was decided according to three predefined criteria: (i) cumulative explained variance exceeding 90%; (ii) each individual principal component contributing less than 5% of the variance; and (iii) an incremental variance difference between successive principal components of less than 0.1%. A shared nearest‐neighbor graph was built based on the selected principal components, followed by unsupervised clustering using the Louvain algorithm. 2D embedding was generated via Uniform Manifold Approximation and Projection (UMAP). Dot plots were used to visualize gene expression patterns across the identified clusters.

### 4.3 Bulk RNA Sequencing and Data Analysis

Total RNA was extracted using TRIzol reagent (Invitrogen, CA, USA) following the manufacturer's protocol. RNA purity and concentration were measured with a NanoDrop 2000 spectrophotometer (Thermo Scientific, USA), and RNA integrity was assessed using an Agilent 2100 Bioanalyzer (Agilent Technologies, Santa Clara, CA, USA). Sequencing libraries were prepared with the VAHTS Universal V10 RNA‐seq Library Prep Kit (Premixed Version) according to the manufacturer's instructions. Transcriptome sequencing and analysis were performed by OE Biotech Co., Ltd. (Shanghai, China). The libraries were sequenced on an Illumina NovaSeq X Plus platform, generating 150 bp paired‐end reads. Raw reads in fastq format were first processed with fastp to remove low‐quality reads, yielding clean reads for each sample. Clean reads were aligned to the reference genome using HISAT2. FPKM (Fragments Per Kilobase Million) values for each gene were calculated, and read counts were obtained using HTSeq‐count. Principal component analysis (PCA) was conducted with R (v3.2.0) to assess sample reproducibility. Differential expression analysis was performed using DESeq2, with significantly differentially expressed genes (DEGs) defined as those with a Q value < 0.05 and foldchange > 2 or foldchange < 0.5. Hierarchical clustering analysis of DEGs was carried out in R (v3.2.0) to visualize gene expression patterns across different groups and samples. A radar chart of the top 30 DEGs was plotted using the R package *ggradar* to display up‐ and down‐regulated genes. Enrichment analyses of DEGs for GO terms, KEGG pathways, Reactome, and WikiPathways were performed based on the hypergeometric distribution, with significant terms identified using R (v3.2.0). Results were visualized via bar plots, chord diagrams, and bubble plots. Gene Set Enrichment Analysis (GSEA) was conducted using GSEA software. This method ranks genes based on their differential expression between two sample groups and evaluates whether a predefined gene set is enriched at the extremes of the ranked list.

### 4.4 Metabolomics

Aspirate the cell culture medium, remove the culture medium, quickly wash 2–3 times with pre‐cooled PBS solution, then add a small amount of PBS solution, gently scrape off the cells with a cell scraper, transfer to a centrifuge tube, centrifuge at 4°C at 300–500 g for 5 min, discard the supernatant, wash with pre‐cooled PBS solution, centrifuge at 4°C at 1000 g for 5 min at low speed, discard the supernatant, wash again with PBS solution, and count the cell suspension containing PBS. Take 1×10^7^ cells from each sample into a 2 mL sterile centrifuge tube, centrifuge at 4°C at 1000 g for 10 min at low speed, and discard the supernatant. Subsequent bioinformatics analysis was then performed by Wuhan Maiwei Metabolism Company.

### 4.5 Animals

Renal tubular epithelium‐specific G6pc knockout (G6PCcKO) mice were generated by crossing male Cdh16‐Cre mice (T007046, GemPharmatech Inc, China) with female G6pc^fl/fl^ mice (T008830, GemPharmatech Inc, China) on a C57BL/6J genetic background. All animal procedures were approved by the Ethics Committee of Zhongnan Hospital, Wuhan University, and conformed to the National Institutes of Health Guide for the Care and Use of Laboratory Animals. Mice were randomly assigned to different experimental groups using a computer‐generated random number sequence prior to the commencement of the treatment period. To establish a calcium oxalate (CaOx) nephrocalcinosis model, mice received daily intraperitoneal injections of glyoxylate (GLY; 100 or 125 mg/kg) for 7 days. Concurrently, the following compounds were administered intraperitoneally for 7 days: 2‐deoxy‐D‐glucose (2‐DG, HY‐13966, MedChemExpress, 500 mg/kg, purity: 99.93%, dissolved in PBS), lactate (HY‐B2227, MedChemExpress, 500 mg/kg, purity: 85.12%, dissolved in a mixture of DMSO, PEG300, Tween‐80, and saline), and SB‐431542 (HY‐10431, MedChemExpress, 50 mg/kg, purity: 99.85%, dissolved in a mixture of DMSO, PEG300, Tween‐80, and saline). At the endpoint, blood and kidney tissues were collected. Blood was drawn from the retro‐orbital plexus, and serum levels of creatinine and blood urea nitrogen (BUN) were measured using commercial assay kits (Bioswamp, China) according to the manufacturer's protocols. All histological scoring, crystal quantification, and image analyses were performed by investigators blinded to the group allocation.

### 4.6 Isolation and Culture of Primary Mouse Renal Proximal Tubular Epithelial Cells (PTECs)

Renal proximal tubular epithelial cells (PTECs) were isolated from 2‐week‐old male C57BL/6 mice. After cervical dislocation and dorsal surface disinfection (75% ethanol), kidneys were aseptically removed. The renal cortex was isolated, minced, and washed by centrifugation in ice‐cold D‐HBSS. Digestion was performed in 0.1% Type IV collagenase for 30 min at 37°C with agitation. The resulting suspension was sequentially filtered through 250 and 80 µm strainers. Following centrifugation, the cell pellet was resuspended in complete DMEM/F12 medium (supplemented with 10% FBS, ITS‐A, antibiotics, NEAA, EGF, and hydrocortisone) and seeded into a 75 cm^2^ flask. Cultures were maintained at 37°C and 5% CO_2_. The initial medium change was conducted at 48 h, with subsequent changes every other day.

### 4.7 Cell Culture

HK‐2 cells were procured from the China Center for Type Culture Collection (Wuhan, China) and confirmed to be free of mycoplasma contamination. The cells were cultured in 1640 medium supplemented with 10% fetal bovine serum (Gibco, USA) and maintained in an incubator (Thermo Fisher Scientific, USA) set at 37°C with 5% CO2 and appropriate humidity.

### 4.8 Cell Transfection

Stable G6PC‐overexpressing and G6PC/LDHA/SNAIL1‐knockdown HK‐2 cell lines were established via lentiviral transduction. Lentiviruses were packaged in HEK293T cells by co‐transfecting transfer and packaging plasmids using Lipo3000. Viral supernatants were harvested at 72 h, filtered, and used to infect HK‐2 cells, followed by selection with 1 µg/mL puromycin. shRNA sequences are listed in Table S1. Transient transfections of human or mouse‐specific siRNAs (GenePharma) were carried out using Lipofectamine 3000, following the manufacturer's protocol. All siRNA sequences are detailed in Table S2.

### 4.9 Renal CaOx Crystals Detection

Paraffin‐embedded renal sections were prepared for the assessment of calcium oxalate (CaOx) crystals. Crystals were visualized by polarized light microscopy on HE‐stained sections and by Pizzolato staining under standard bright‐field microscopy. The extent of crystal deposition was quantified using ImageJ software.

### 4.10 Assessment of Tubular Injury

Tubular injury was evaluated on periodic acid‐Schiff (PAS)‐stained kidney sections and scored based on the percentage of affected tubules, as previously described.

### 4.11 Immunohistochemistry (IHC)

For immunohistochemical (IHC) staining, kidney sections were incubated overnight at 4°C with the following primary antibodies: anti‐G6PC (1:50, 29084‐1‐AP, proteintech, Wuhan, China), anti‐PC (1:200, 16588‐1‐AP, proteintech), anti‐PCK1 (1:200, 66862‐1‐Ig, proteintech), anti‐FBP1 (1:200, 12842‐1‐AP, proteintech), anti‐PKM (1:250, 10078‐2‐AP, proteintech), and anti‐HK1 (1:500, 68419‐1‐Ig, proteintech). Images of the renal cortex were captured using an Olympus microscope. The relative protein expression levels were quantified from the cortical images using ImageJ software.

### 4.12 Immunofluorescence

HK‐2 and PTEC cells were seeded onto glass coverslips and cultured for 24 h. After treatment, the medium was removed and the cells were fixed with paraformaldehyde, washed with PBS, and blocked with 3% BSA. Next, the cells were incubated overnight with anti‐E‐cadherin (1:100, 60335‐1‐Ig, proteintech), anti‐α‐SMA (1:100, ab46545, Abcam, USA), and fluorescently labeled secondary antibodies for 2 h. Kidney sections were incubated overnight with anti‐G6PC (1:50, 29084‐1‐AP, proteintech), anti‐PC (1:50, 16588‐1‐AP, proteintech), anti‐PCK1 (1:400, 66862‐1‐Ig, proteintech), anti‐FBP1 (1:200, 12842‐1‐AP, proteintech), anti‐PKM (1:200, 10078‐2‐AP, proteintech), and anti‐HK1 (1:1024, 68419‐1‐Ig, proteintech), anti‐E‐cadherin (1:200, 60335‐1‐Ig, proteintech), anti‐α‐SMA (1:50, 14395‐1‐AP, proteintech), anti‐AQP1 (1:200, 66805‐1‐Ig, proteintech). Following DAPI staining, images were captured using a fluorescence microscope (Olympus IX71, Japan).

### 4.13 HE Staining

Following deparaffinization in xylene and rehydration through a graded ethanol series, sections were stained with hematoxylin and eosin (HE) using a standard protocol. Briefly, hematoxylin staining (3–5 min) was followed by bluing, eosin counterstaining (5 min), dehydration, clearing in xylene, and mounting with neutral resin. Images were acquired using a Nikon Eclipse E100 microscope.

### 4.14 PAS Staining

The samples were deparaffinized with xylene and ethanol, stained with periodic acid, Schiff's reagent, and hematoxylin, dehydrated with anhydrous ethanol, cleared with xylene, and mounted with neutral resin in turn. The images were captured and analyzed using a Nikon Eclipse E100 microscope.

### 4.15 MASSON Staining

The samples were deparaffinized with xylene and ethanol, soaked in potassium, stained with iron hematoxylin, Ponceau Acid Fuchsin, phosphomolybdic acid, aniline blue, differentiated with 1% glacial acetic acid, dehydrated, cleared with xylene, and mounted with neutral resin in turn. The images were captured and analyzed using a Nikon Eclipse E100 microscope.

### 4.16 Sirius Red Staining

The specimens were deparaffinized with xylene and ethanol, rinsed with water, stained with Sirius Red solution, and mounted with neutral resin in turn. Images were captured and analyzed using a Nikon Eclipse E100 microscope.

### 4.17 Lactate Assay Experiment Protocol

Fresh tissues or cell suspensions were homogenized and the supernatant were retained. Total protein concentrations in supernatants were determined by BCA assay (Thermo Fisher) to normalize lactate levels (expressed as µmol/g protein or µmol/10^6^ cells). L‐Lactate concentrations in kidney tissues, and cell supernatants were quantified using the L‐Lactate (LA) Colorimetric Assay Kit (Elabscience, E‐BC‐K044‐M, China) based on enzymatic colorimetric principles in accordance with the manufacturers’ protocols.

### 4.18 Measurement of Glucose‐6‐Phosphate, Fructose‐6‐Phosphate, and Pyruvate Levels

Fresh tissues or cell suspensions were homogenized and the supernatant were retained. Glucose‐6‐phosphate, fructose‐6‐phosphate, and pyruvate concentrations were quantified using the Glucose‐6‐Phosphate Assay Kit (Beyotime, S0185, China), Fructose‐6‐Phosphate Assay Kit (Beyotime, S0183S, China), and Amplex Red Pyruvate Assay Kit (Beyotime, S0299S, China) based on absorbance in accordance with the manufacturers’ protocols.

### 4.19 Prediction of Lactylation Site

We use the DeepKlapred database to analyze the most probable site for lactylation of Snail1. We apply a combination of token embedding and position embedding to convert protein sequences into numerical feature representations. The sequence embeddings are then processed through a BiGRU‐Transformer architecture, which captures both local and global dependencies within the sequence, providing a richer representation of the underlying biological context. In addition to sequence embedding, we incorporate six sequence descriptors (QSOrder, CTDC, CTDT, CTDD, DistancePair, and DDE) to further enhance extraction process. Finally, we utilize a cross‐attention fusion mechanism to integrate the sequence embedding with descriptor‐based features, allowing the model to capture complex interactions between different feature representations [38]. After submitting the sequence, the prediction results are displayed in an intuitive format, showing the predicted Kla sites and the prediction probability given by the model.

### 4.20 Quantitative PCR (qPCR)

Total RNA was isolated from mouse kidneys and HK2 cells utilizing the trizol reagent (Invitrogen, USA) and subsequently reverse transcribed into complementary DNA (cDNA) using the HiScript III Reverse Transcriptase (Vazyme, China). Quantitative PCR (qPCR) was conducted employing the Taq Pro Universal SYBR qPCR Master Mix (Vazyme, China) in accordance with the manufacturer's instructions. The specific primers employed in this study are detailed in Table S3.

### 4.21 Immunoblotting

Total protein from mouse kidneys, HK‐2 cells, and primary PTEC cells was extracted with RIPA lysis buffer (Servicebio, China), and the protein concentration was measured using the BCA assay (Beyotime Biotechnology, China). The primary antibodies used were as follows: anti‐G6PC (1:1000, 40 kDa, 29084‐1‐AP, proteintech), anti‐PC (1:1000, 125 kDa, 16588‐1‐AP, proteintech), anti‐PCK1 (1:1000, 69 kDa, 66862‐1‐Ig, proteintech), anti‐FBP1 (1:1000, 37 kDa, 12842‐1‐AP, proteintech), anti‐PKM (1:1000, 58 kDa, 10078‐2‐AP, proteintech), and anti‐HK1 (1:5000, 100 kDa, 68419‐1‐Ig, proteintech), anti‐E‐cadherin (1:1000, 120 kDa, 60335‐1‐Ig, proteintech), anti‐β‐catenin (1:1000, 92 kDa, 51067‐2‐AP, proteintech), anti‐N‐cadherin (1:1000, kDa, 22018‐1‐AP, proteintech), anti‐Vimentin (1:1000, 56 kDa, 10366‐1‐AP, proteintech, China), anti‐α‐SMA (1:1000, 42 kDa, 14395‐1‐AP, proteintech), anti‐TGF‐β (1:1000, 42 kDa, 26155‐1‐AP, proteintech), anti‐Snail1 (1:1000, 36 kDa, 13099‐1‐AP, proteintech), anti‐ACTIN (1:5000, 42 kDa, 66009‐1‐Ig, proteintech), and anti‐GAPDH (1:5000, 36 kDa, 10494‐1‐AP, proteintech).

### 4.22 Immunoprecipitation

For co‐immunoprecipitation, 200 µg of protein lysates were immunoprecipitated overnight at 4°C using anti‐Snail1 or anti‐pan‐Kla antibody, followed by a 4‐hour incubation with Protein A/G beads. The washed precipitates were boiled in SDS buffer and analyzed by immunoblotting with the specified antibodies: anti–pan‐Kla antibody (1:1000, PTM‐1401RM, PTM Biolabs Inc, China), anti‐CBP antibody (1:1000, 125 kDa, 16588‐1‐AP, proteintech), anti‐p300 antibody (1:1000, 300 kDa, 20695‐1‐AP, proteintech), or anti‐SNAIL1 (1:1000, 36 kDa, 13099‐1‐AP, proteintech), respectively.

### 4.23 Chromatin Immunoprecipitation (ChIP)

The binding of SNAIL1 to the TGF‐β promoter was assessed by chromatin immunoprecipitation (ChIP). After treating HK‐2 cells with 300 µg/mL COM or 20 µm lactate for 24 h, proteins were cross‐linked to DNA with formaldehyde. The sonicated chromatin was immunoprecipitated with an anti‐Snail1 antibody, and the co‐precipitated DNA was analyzed by qPCR to determine promoter enrichment relative to an IgG control.

### 4.24 Statistical Analysis

Data are expressed as mean ± SD from at least three independent replicates. Statistical significance was determined using two‐tailed Student's t‐tests or ANOVA in GraphPad Prism (v8.0), with *p < 0.05 considered significant (*p < 0.05, **p < 0.01, ***p < 0.001, ****p < 0.0001). Single‐cell data were analyzed using the “Seurat” package in R.

### 4.25 Ethic Statements

The animal study was approved by the Experimental Animal Welfare Ethics Committee at Zhongnan Hospital of Wuhan University (approval number: MRI2025‐LAC052). For human study: not applicable.

## Author Contributions

Kai Liu and Boming Zhang contributed equally to this work. All authors contributed to this work and approved the submitted version. Kai Liu, Ruixin Fan, Chen Duan, Heng Li, and H.X. conceived the project and designed the study. Kai Liu, Boming Zhang, Ruixin Fan performed the experiments. Yangjun Zhang, Huahui Wu, Xiangyang Yao, Xiongmin Mao, Bo Li, Youmiao Zeng, and Zhenzhen Xu analyze the data. Kai Liu and Boming Zhang wrote the manuscript. Heng Li and Hua Xu improved the manuscript.

## Funding

This work was supported by National Natural Science Foundation of China (82270803 and 82070726), Natural Science Foundation of Hubei Province (2024AFA019), Hubei Health Committee (WSJKRC2024009), Taikang Life Medicine Center (CXTQSYS2023003), Science, Technology and Innovation Seed Fund of Zhongnan Hospital of Wuhan University (CXPY2022088), Translational Medicine and Interdisciplinary Research Joint Fund of Zhongnan Hospital of Wuhan University (ZNJC2022010), Medical Science and Technology Innovation Platform Construction Support Project of Zhongnan Hospital of Wuhan University (PTXM2022006), and Technology Achievement Transformation Fund of Zhongnan Hospital of Wuhan University (LCYFZD2024002).

## Conflicts of Interest

None of the authors have a conflict of interest to disclose.

## Supporting information




**Supporting file**: advs75585‐sup‐0001‐SuppMat.docx

## Data Availability

The data that support the findings of this study are available from the corresponding author upon reasonable request.
