# Supplementary material for: G6PC Downregulation Promotes Renal Calcium Oxalate Stone Formation via Lactate‐Induced SNAIL1 K206 Lactylation and Epithelial‐Mesenchymal Transition
Source: Adv Sci (Weinh). 2026 May 10;13(43):e75585. doi: 10.1002/advs.75585 (PMC13335882; doi:10.1002/advs.75585)
Supplement: Supplementary file 1 — Supporting file: advs75585‐sup‐0001‐SuppMat.docx [file ADVS-13-e75585-s001.docx]

**Supplementary Figure 1.** Single-cell profiling reveals altered glucose metabolism in proximal tubular cells during renal calcification.

(A-B) Mouse calcium oxalate nephrocalcinosis model.

(A) Cellular cluster composition.

(B) Spatial transcriptomics showing renal G6PC distribution in Control and Gly-treated groups.

(C-G) Human Randall’s plaque versus adjacent normal renal tissue.

(C) UMAP visualization of 15 distinct cell types.

(D) Cellular cluster composition.

(E) Volcano plot of differentially expressed genes (DEGs).

(F) UMAP plots showing subcluster distribution (left) and G6PC expression (right).

(G) KEGG pathway enrichment analysis of DEGs in PTECs.

**Supplementary Figure 2.** Expression patterns of glycolytic and gluconeogenic marker genes across cell subclusters in Control versus Gly-treated groups.

(A–J) For each gene, UMAP visualization shows its distribution across subclusters (left panels), while violin plots illustrate expression levels in Control and Gly groups (right panels). (A, B) Pc; (C, D) Pck1; (E, F) Fbp1; (G, H) Hk1; (I, J) Pkm.

**Supplementary Figure 3.** Expression distribution of glycolytic and gluconeogenic marker genes across cell subclusters in human Randall’s plaque versus adjacent normal renal tissue.

(A–J) For each gene, UMAP visualization shows its distribution across subclusters (left panels), while bubble plots illustrate expression levels in Randall’s plaque (Randalls) and adjacent normal tissue (Renalap) (right panels). (A, B) Pc；(C, D) Pck1；(E, F) Fbp1；(G, H) Hk1；(I, J) Pkm.

**Supplementary Figure 4.** G6PC emerged as the most downregulated gluconeogenic gene in models of calcium oxalate nephrolithiasis.

(A) Relative mRNA levels of PC, PCK1, FBP1, and G6PC in HK-2 cells treated with different concentrations of COM (n = 3 per group).

(B) Relative mRNA levels of PC, PCK1, FBP1, and G6PC in PTECs treated with different concentrations of COM (n = 3 per group).

(C) Relative mRNA levels of PC, PCK1, FBP1, and G6PC in mice treated with different concentrations of Gly (n = 3 per group).

(D) Relative mRNA levels of PC, PCK1, FBP1, and G6PC in mice treated with oxalate (n = 3 per group).

Error bars show the mean ± SD. **p* < 0.05, ***p* < 0.01, ****p* < 0.001, *****p* < 0.0001. The P value was determined using one-way ANOVA (A-C) or two-tailed unpaired Student’s test (D). All numbers (n) are biologically independent experiments.

**Supplementary Figure 5.** Decreased gluconeogenesis and increased glycolysis in kidney stone models.

(A-B) Immunoblots of the protein expression levels and quantification of gluconeogenic and glycolytic enzyme in mice treated with (A) different concentrations of Gly. (B) Oxalate (n = 3 per group).

(C-D) Immunofluorescence images showing the gluconeogenic and glycolytic enzyme in kidney tissues from kidney stone models (scale bar = 50 μm, n = 6 per group).

(E-F) Immunohistochemistry images showing the gluconeogenic and glycolytic enzyme in kidney tissues from kidney stone models (scale bar = 50 μm, n = 6 per group).

Error bars show the mean ± SD. **p* < 0.05, ***p* < 0.01, ****p* < 0.001, *****p* < 0.0001. The P value was determined using one-way ANOVA (A) or two-tailed unpaired Student’s test (B). All numbers (n) are biologically independent experiments.

**Supplementary Figure 6.** Comprehensive analysis of gluconeogenic metabolites in CaOx stone models.

(A-C) Glucose-6-phosphate levels in (A, B) kidneys from Gly or oxalate-treated mice and (C) in COM-treated HK-2 cells or PTECs (n = 6 per group).

(D-E) Glucose-6-phosphate levels in G6PC-knockdown and G6PC-overexpressing (D) HK-2 cell or (E) PTECs (n = 6 per group).

(F-H) Fructose-6-phosphate levels in (F, G) kidneys from Gly or oxalate-treated mice and (H) in COM-treated HK-2 cells or PTECs (n = 6 per group).

(I-J) Fructose-6-phosphate levels in G6PC-knockdown and G6PC-overexpressing (I) HK-2 cell or (J) PTECs (n = 6 per group).

(K-M) Pyruvate levels in (K, L) kidneys from Gly or oxalate-treated mice and (M) in COM-treated HK-2 cells or PTECs (n = 6 per group).

(N-O) Pyruvate levels in G6PC-knockdown and G6PC-overexpressing (N) HK-2 cell or (O) PTECs (n = 6 per group).

Error bars show the mean ± SD. **p* < 0.05, ***p* < 0.01, ****p* < 0.001, *****p* < 0.0001. The P value was determined using one-way ANOVA (A, C-F, H-K, M-O) or two-tailed unpaired Student’s test (B, G, L). All numbers (n) are biologically independent experiments.

**Supplementary Figure 7.** Gly induces the EMT of kidney stone models.

(A-J) Representative images of (A, F) HE staining by polarized light optical microscopy and quantification of the proportion of crystal deposition area (scale bar = 500μm, n = 6 per group);

(B, G) Von Kossa staining and quantification of the proportion of calcium oxalate crystal deposition area (scale bar = 20μm, n = 6 per group);

(C, H) PAS staining and tubular injury score (scale bar = 20μm, n = 6 per group);

(D, I) Sirus Red staining and quantification of the proportion of fibrotic area (scale bar = 20μm, n = 6 per group);

(E, J) Masson staining and quantification of the proportion of fibrotic area (scale bar = 20μm, n = 6 per group) in kidney tissues from mice treated with different concentrations of Gly.

(K-L) Serum BUN and creatinine levels of mice treated with different concentrations of Gly (n = 6 per group).

(M-O) Immunofluorescence images and quantification of the E-cadherin (red) and α-SMA (green) levels in kidney tissues from mice treated with different concentrations of Gly (scale bar = 50 μm, n = 6 per group).

Error bars show the mean ± SD. **p* < 0.05, ***p* < 0.01, ****p* < 0.001, *****p* < 0.0001. The P value was determined using one-way ANOVA (F-L, N and O). All numbers (n) are biologically independent experiments.

**Supplementary Figure 8.** Oxalate induces the EMT of kidney stone models.

(A-J) Representative images of (A, F) HE staining by polarized light optical microscopy and quantification of the proportion of crystal deposition area (scale bar = 500 μm, n = 6 per group);

(B, G) Von Kossa staining and quantification of the proportion of calcium oxalate crystal deposition area (scale bar = 20μm, n = 6 per group);

(C, H) PAS staining and tubular injury score (scale bar = 20μm, n = 6 per group);

(D, I) Sirus Red staining and quantification of the proportion of fibrotic area (scale bar = 20 μm, n = 6 per group);

(E, J) Masson staining and quantification of the proportion of fibrotic area (scale bar = 20μm, n = 6 per group) in kidney tissues from mice treated with oxalate.

(K-L) Serum BUN and creatinine levels of mice treated with oxalate (n = 6 per group). (M) Immunoblots of the protein expression levels and quantification of EMT-related markers in kidney tissues from mice treated with oxalate (n = 3 per group).

(N-P) Immunofluorescence images and quantification of the E-cadherin (red) and α-SMA (green) levels in kidney tissues from mice treated with oxalate (scale bar = 50 μm, n = 6 per group).

Error bars show the mean ± SD. **p* < 0.05, ***p* < 0.01, ****p* < 0.001, *****p* < 0.0001. The P value was determined using two-tailed unpaired Student’s test (F-M, O and P). All numbers (n) are biologically independent experiments.

**Supplementary Figure 9.** COM induces the EMT in cellular models of kidney stone disease.

(A) Immunoblots of the protein expression levels and quantification of EMT-related markers in HK-2 cells treated with different concentrations of COM (n = 4 per group).

(B-D) Cell immunofluorescence images and quantification of the E-cadherin (red) and α-SMA (green) levels in HK-2 cells treated with different concentrations of COM (scale bar = 50μm, n = 3 per group).

(E) Immunoblots of the protein expression levels and quantification of EMT-related markers in PTEC cells treated with different concentrations of COM (n = 4 per group).

(F-H) Cell immunofluorescence images and quantification of the E-cadherin (red) and α-SMA (green) levels in PTEC cells treated with different concentrations of COM (scale bar = 50 μm, n = 3 per group).

Error bars show the mean ± SD. **p* < 0.05, ***p* < 0.01, ****p* < 0.001, *****p* < 0.0001. The P value was determined using one-way ANOVA (A, C-E, G and H). All numbers (n) are biologically independent experiments.

**Supplementary Figure 10.** Construction of G6PC knockdown and overexpression cell lines in HK-2 and PTECs.

(A-F) Construction of G6PC knockdown and overexpression cell lines in HK-2 cell.

(A) Immunoblotting of G6PC protein level in HK-2 cell following G6PC overexpression (n = 3 per group).

(B-C) Quantification of G6PC protein and mRNA levels in HK-2 cell following G6PC overexpression (n = 3 per group).

(D) Immunoblotting of G6PC protein level in HK-2 cell following G6PC knockdown (n = 3 per group).

(E-F) Quantification of G6PC protein and mRNA levels in HK-2 cell following G6PC knockdown (n = 3 per group).

(G-L) Construction of G6PC knockdown and overexpression cell lines in PTECs.

(G) Immunoblotting of G6PC protein level in PTECs following G6PC overexpression (n = 3 per group).

(H-L) Quantification of G6PC protein and mRNA levels in PTECs following G6PC overexpression (n = 3 per group).

(J) Immunoblotting of G6PC protein level in PTECs following G6PC knockdown (n = 3 per group).

(K-L) Quantification of G6PC protein and mRNA levels in PTECs following G6PC knockdown (n = 3 per group).

Error bars show the mean ± SD. **p* < 0.05, ***p* < 0.01, ****p* < 0.001, *****p* < 0.0001. The P value was determined using two-tailed unpaired Student’s test (B, C, H and I) or one-way ANOVA (E, F, K and L). All numbers (n) are biologically independent experiments.

**Supplementary Figure 11.** Generation and genotyping of Cre^+^/G6PC^fl/fl^ mice.

(A) Breeding scheme for Cre^+^/G6PC^fl/fl^ mice. G6PC^fl/fl^ homozygotes were obtained from G6PC^fl/−^ heterozygotes, then crossed with Gpt-Cdh16-CreERT2 mice. Offspring were screened for G6PC^fl/−^Cdh16-Cre, which were subsequently mated with G6PC^fl/fl^ mice to obtain Cre^+^/G6PC^fl/fl^ mice. Tamoxifen was administered at appropriate weeks to induce Cre recombinase activity, yielding the target Cre^+^/G6PC^fl/fl^ mice.

(B–D) Genotyping results.

(B) G6PC^fl/fl^ genotyping: WT band = 325 bp, FL band = 406 bp.

(C) Cdh16-Cre knock‑in (KI) band = 355 bp.

(D) Cdh16‑Cre wild‑type (WT) band = 302 bp; KI band = 2336 bp. B6 genomic DNA served as negative control; N = no‑template control.

(E) Immunofluorescence staining of E‑cadherin (red) and G6PC (green) in kidney sections, with DAPI (blue) marking nuclei (Scale bar = 50 µm, n = 6 per group).

All numbers (n) are biologically independent experiments.

**Supplementary Figure 12.** Validation of proximal tubule-specific G6PC expression and its efficient deletion by Cdh16-Cre.

(A) Immunoblotting of G6PC protein level in Cre^-^/G6PC^fl/fl^ mice and Cre^+^/G6PC^fl/fl^ mice (n = 4 per group).

(B) Immunofluorescence staining of AQP1 (red) and Cdh16 (green) in kidney sections of Cre^+^/G6PC^fl/fl^ mice, with DAPI (blue) marking nuclei (Scale bar = 50 µm, n = 6 per group).

(C) Immunofluorescence staining showed that G6PC was deleted in proximal tubule from Cre^+^/G6PC^fl/fl^ mice (Scale bar = 50 µm, n = 6 per group). Aquaporin-1 (AQP1) was used as a proximal tubular marker. Dolichos biflorus agglutinin (DBA) was used as a marker for collecting duct.

All numbers (n) are biologically independent experiments.

**Supplementary Figure 13.** Knockdown of G6PC induces EMT, whereas its overexpression attenuates COM-induced EMT in PTEC cells.

(A) Immunoblots of the protein expression levels and quantification of EMT-related markers in PTEC cells transfected with G6PC-siRNA (n = 3 per group).

(B-D) Cell immunofluorescence images and quantification of the E-cadherin (red) and α-SMA (green) levels in PTEC cells transfected with G6PC-siRNA (scale bar = 50 μm, n = 3 per group).

(E) Immunoblots of the protein expression levels and quantification of EMT-related markers in PTEC cells transfected with G6PC overexpression plasmid (n=3 per group).

(F-H) Cell immunofluorescence images and quantification of the E-cadherin (red) and α-SMA (green) levels in PTEC cells transfected with G6PC overexpression plasmid (scale bar = 50 μm, n = 3 per group).

Error bars show the mean ± SD. **p* < 0.05, ***p* < 0.01, ****p* < 0.001, *****p* < 0.0001. The P value was determined using one-way ANOVA (A, C-E, G and H). All numbers (n) are biologically independent experiments.

**Supplementary Figure 14.** Lactate induces EMT in PTEC cells.

(A) Immunoblots of the protein expression levels and quantification of EMT-related markers in PTEC cells treated with different concentrations of lactate (n = 4 per group).

(B-D) Cell immunofluorescence images and quantification of the E-cadherin (red) and α-SMA (green) levels in PTEC cells treated with different concentrations of lactate (scale bar = 50 μm, n = 3 per group).

Error bars show the mean ± SD. **p* < 0.05, ***p* < 0.01, ****p* < 0.001, *****p* < 0.0001. The P value was determined using one-way ANOVA (A, C and D). All numbers (n) are biologically independent experiments.

**Supplementary Figure 15.** TGF-β/SMAD3 signaling is activated in kidney stone models.

(A-D) The relative mRNA levels of SMAD2 and SMAD3 in kidney tissues from mice treated with (A and B) Gly or (C and D) Oxalate (n = 3 per group).

(E-F) Immunoblots and quantification of the phosphorylation of SMAD3 in kidney tissues from mice treated with (E) Gly or (F) Oxalate (n = 3 per group).

(G-J) The relative mRNA levels of SMAD2 and SMAD3 in (G and H) HK-2 or (I and J) PTEC cells treated with COM (n = 3 per group).

(K-L) Immunoblots and quantification of the phosphorylation of SMAD3 in (K) HK-2 or (L) PTEC cells treated with COM (n = 4 per group).

(M) TGF-β/SMAD3 signaling in G6PCcKO mice (n = 4 per group).

Error bars show the mean ± SD. **p* < 0.05, ***p* < 0.01, ****p* < 0.001, *****p* < 0.0001. The P value was determined using two-tailed unpaired Student’s test (C, D, F and M) or one-way ANOVA (A, B, E, G-L). All numbers (n) are biologically independent experiments.

**Supplementary Figure 16.** Lactate activates TGF-β/SMAD3 signaling in HK-2 cells or PTEC cells.

(A-D) The relative mRNA levels of SMAD2 and SMAD3 in (A and B) HK-2 cells or (C and D) PTEC cells (n = 3 per group).

(E-F) Immunoblots of the protein expression levels and quantification of TGF-β/SMAD3 signaling in (E) HK-2 cells or (F) PTEC cells treated with different concentrations of lactate (n = 4 per group).

Error bars show the mean ± SD. **p* < 0.05, ***p* < 0.01, ****p* < 0.001, *****p* < 0.0001. The P value was determined using one-way ANOVA (A-F). All numbers (n) are biologically independent experiments.

**Supplementary Figure 17.** Snail2, Twist1, and Zeb1 are not involved in EMT induction.

(A–U) Immunoblots of the protein expression levels and quantification of EMT-related markers of the indicated transcription factors in HK-2 cells treated with COM crystals or lactate (n = 3 per group).

Error bars show the mean ± SD. **p* < 0.05, ***p* < 0.01, ****p* < 0.001, *****p* < 0.0001. The P value was determined using one-way ANOVA (D-U). All numbers (n) are biologically independent experiments.

**Supplementary Figure 18.** Construction of LDHA knockdown cell lines in HK-2 and PTECs.

(A) Immunoblotting of LDHA protein level in HK-2 cell following LDHA knockdown (n = 3 per group).

(B-C) Quantification of LDHA protein and mRNA levels in HK-2 cell following LDHA knockdown (n = 3 per group).

(D) Immunoblotting of LDHA protein level in PTECs following LDHA knockdown (n = 3 per group).

(E-F) Quantification of LDHA protein and mRNA levels in PTECs following LDHA knockdown (n = 3 per group).

Error bars show the mean ± SD. **p* < 0.05, ***p* < 0.01, ****p* < 0.001, *****p* < 0.0001. The P value was determined using one-way ANOVA (B, C, E and F). All numbers (n) are biologically independent experiments.

**Supplementary Figure 19.** Reducing intracellular lactate levels attenuates COM-induced changes in EMT markers.

(A) Immunoblots of the protein expression levels and quantification of EMT-related markers in PTECs treated with 2-DG and COM (n = 3 per group).

(B-D) Cell immunofluorescence images and quantification of the E-cadherin (red) and α-SMA (green) levels in PTECs treated with 2-DG and COM (scale bar = 50 μm, n = 3 per group).

(E) Immunoblots of the protein expression levels and quantification of EMT-related markers in LDHA knockdown PTECs treated with COM (n = 4 per group).

(F-H) Cell immunofluorescence images and quantification of the E-cadherin (red) and α-SMA (green) levels in LDHA knockdown PTECs treated with COM (scale bar = 50 μm, n = 3 per group).

Error bars show the mean ± SD. **p* < 0.05, ***p* < 0.01, ****p* < 0.001, *****p* < 0.0001. The P value was determined using one-way ANOVA (A, C-E, G and H). All numbers (n) are biologically independent experiments.

**Supplementary Table 1. Sequence of shRNA**

| Name | Vector | Restriction Site | Target Sequence（5’-3’） |
| --- | --- | --- | --- |
| h*G6PC1-1 | pLKO.1-copGFP-PURO | AgeI，EcoRI | GCCGCACAAGAAGTCGTTGTA |
| h*G6PC1-2 |  |  | CGCACAAGAAGTCGTTGTAAG |
| h*G6PC1-3 |  |  | TTCGGTGCTTGAATGTCATTT |
| h*G6PC1-4 |  |  | CGTCTTTAAGTGGATTCTCTT |

**Supplementary Table 2. Sequence of siRNA**

| Name | Species | （5’-3’） | Sequence（5’-3’） |
| --- | --- | --- | --- |
| G6pc-1 | mouse | sense | GGAUCUUCCUGCUACCUAUTT |
| G6pc-1 | mouse | antisense | AUAGGUAGCAGGAAGAUCCTT |
| G6pc-2 | mouse | sense | CCACAAAGGUGCUGAAUUUTT |
| G6pc-2 | mouse | antisense | AAAUUCAGCACCUUUGUGGTT |
| G6pc-3 | mouse | sense | GCCUGUUGUUUCCAGAAUATT |
| G6pc-3 | mouse | antisense | UAUUCUGGAAACAACAGGCTT |

**Supplementary Table 3. qPCR Primer Sequences**

| Primer | Species | （5’-3’） | Sequence（5’-3’） |
| --- | --- | --- | --- |
| PC | human | Forward | ACAGAGGTGAGATTGCCATCC |
|  | human | Reverse | CACTGCATCTACGTTGTTCTCC |
| FBP1 | human | Forward | CGCGCACCTCTATGGCATT |
|  | human | Reverse | TTCTTCTGACACGAGAACACAC |
| G6pc | human | Forward | GTGTCCGTGATCGCAGACC |
|  | human | Reverse | GACGAGGTTGAGCCAGTCTC |
| HK1 | human | Forward | GCTCTCCGATGAAACTCTCATAG |
|  | human | Reverse | GGACCTTACGAATGTTGGCAA |
| PCK1 | human | Forward | AAAACGGCCTGAACCTCTCG |
|  | human | Reverse | ACACAGCTCAGCGTTATTCTC |
| PKM | human | Forward | ATGTCGAAGCCCCATAGTGAA |
|  | human | Reverse | TGGGTGGTGAATCAATGTCCA |
| ACTIN | human | Forward | GAGAAAATCTGGCACCACACCT |
|  | human | Reverse | GCACAGCCTGGATAGCAACGTA |
| Pc | mouse | Forward | CTGAAGTTCCAAACAGTTCGAGG |
|  | mouse | Reverse | CGCACGAAACACTCGGATG |
| Fbp1 | mouse | Forward | CACCGCGATCAAAGCCATCT |
|  | mouse | Reverse | AGGTAGCGTAGGACGACTTCA |
| G6pc | mouse | Forward | CGACTCGCTATCTCCAAGTGA |
|  | mouse | Reverse | GTTGAACCAGTCTCCGACCA |
| hk1 | mouse | Forward | CGGAATGGGGAGCCTTTGG |
|  | mouse | Reverse | GCCTTCCTTATCCGTTTCAATGG |
| Pck1 | mouse | Forward | CTGCATAACGGTCTGGACTTC |
|  | mouse | Reverse | CAGCAACTGCCCGTACTCC |
| Pkm | mouse | Forward | GCCGCCTGGACATTGACTC |
|  | mouse | Reverse | CCATGAGAGAAATTCAGCCGAG |
| actin | mouse | Forward | AAGACCTCTATGCCAACAC |
|  | mouse | Reverse | CTGCTTGCTGATCCACAT |
